# Hybrid fuzzy C-means and deep learning framework for intelligent fault classification in solar PV systems

**DOI:** 10.3389/frai.2026.1881404

**Published:** 2026-07-23

**Authors:** V. Vignesh, R. Senthil Kumar, G. Suganeshwari

**Affiliations:** 1 School of Electrical Engineering, Vellore Institute of Technology, Chennai, Tamil Nadu, India; 2 School of Computer Science and Engineering, Vellore Institute of Technology, Chennai, Tamil Nadu, India

**Keywords:** deep learning, fault classification, fuzzy c-means, gated recurrent unit, photovoltaic

## Abstract

Photovoltaic (PV) systems have proven themselves to be a viable alternative energy source; however, there are multiple faults related to PV systems which cause energy losses and low efficiencies. Manual or rule-based algorithms are traditionally used for fault diagnosis, which are not efficient and unsuitable for real-time applications. In this paper, a novel hybrid intelligent classification system for PV fault detection is proposed by integrating Fuzzy C-Means (FCM) clustering and Deep Learning (DL) techniques such as Multi-Layer Perceptron (MLP), Long Short-Term Memory (LSTM), Bidirectional LSTM (BiLSTM) and Gated Recurrent Unit (GRU). The dataset consists of 102,400 samples collected from a real-time 10 kW solar PV system operating under varying irradiance conditions ranging from 600 W/m^2^ to 1,000 W/m^2^ and temperature conditions ranging from 25 °C to 40 °C. The FCM technique is used to enhance the extracted features by clustering the membership functions, and the obtained features are used for model training. The performance of proposed models is evaluated using the classification metrics and confusion matrices. The proposed FCM + GRU model achieved 91.13% accuracy 0.79 precision, 0.76 recall, and F1-score of 0.78. The obtained results confirm the effectiveness of the proposed hybrid framework by improving fault classification performance under various operating environments.

## 1 Introduction

The global solar industry is facing an escalating reliability crisis, with equipment failure-induced outages increasing from 3.13% in 2022 to 4.47% in 2023. Such issues lead to massive financial consequences, leading to an average annual loss of roughly $4.6 billion—or about $4,696 per MW (Labiod et al., 2025). The global solar report 2024 finds that global revenue loss due to equipment-driven underperformance last year reached $4.6 billion, and that power loss due to equipment anomalies on solar farms rose from 3.13 to 4.47% year-over-year (Solar Power, 2024). Large-scale PV systems are particularly vulnerable to mismatch faults, partial shading, open-circuit conditions, hotspot formation, and inverter failures, all of which contribute to reduced energy yield and increased maintenance costs. Consequently, accurate and intelligent fault diagnosis frameworks have become essential for improving system reliability, minimizing downtime, and enhancing the economic sustainability of modern solar power infrastructure (Teta et al., 2024).

In the current context, PV plants have become an integral part of renewable energy facilities, which are capable of addressing the growing worldwide energy demand in an environmentally friendly manner. However, maintaining their efficient and reliable performance is a challenging task while considering the presence of different types of faults and associated risks. The need for implementing sophisticated techniques like Artificial Intelligence (AI) methodologies was demonstrated by previous researchers in (Saravanan et al., 2024; Taghezouit et al., 2024). Meanwhile, the need for fault detection and monitoring solutions is increasing in relation to the increase in complexity and deteriorated performance of PV systems (Taghezouit et al., 2024).

Various faults can occur in PV systems, including Open Circuit (OC), Short Circuit (SC), Ground Fault (GF), and Mismatch Fault (MF), which may significantly reduce the power output and overall efficiency of the PV array (Khalil et al., 2020). Conventional fault diagnosis approaches such as manual inspection and hardware-based measurements are generally accurate but are often time-consuming, computationally expensive, and unsuitable for large-scale PV installations (Rahman et al., 2021). To overcome these limitations, Machine Learning (ML) and DL methods have recently gained considerable attention for automated fault diagnosis applications. These intelligent methods can analyze the large volumes of operational data and identify complex nonlinear relationships among parameters such as voltage, current, power, irradiance, and temperature. The application of AI-based methods for PV fault detection has been comprehensively reviewed in (Thakfan and Bin Salamah, 2024), while studies in (Priyadarshini et al., 2025; Punitha et al., 2024) demonstrated improved monitoring performance using DL and IoT-integrated fault diagnosis systems.

Xu et al. (2022) employed the FCM algorithm for fault identification of PV system using extracted features from I–V curves. Zhao et al. (2018) combined FCM clustering for fault diagnosis and fuzzy membership function to classify the faults. However, the predefined clustering and fuzzy membership rules limit the adaptability of dynamic PV operating situations. Li et al. (2026) developed a hybrid framework integrating unsupervised clustering and statistical methods for PV systems, where FCM was used for statistical features of the PV array operational data to identify PV strings under partial shading conditions. Hence, FCM is incorporated into the proposed framework for efficient clustering of PV fault data and provides meaningful input patterns for subsequent DL-based classification.

Ramachandran et al. (2025) developed a deep learning-based PV fault classification framework using RGB imaging techniques and compared several preprocessing strategies to improve classification performance. A comprehensive review of imaging techniques, preprocessing approaches, and AI-based visual fault diagnosis methods for photovoltaic systems is developed in (Balachandran et al., 2024). In (Ramachandran et al., 2024), the authors investigated machine learning algorithms for renewable energy monitoring applications and demonstrated the capability of intelligent data-driven models for analysing energy system behavior and operational conditions. These studies collectively confirm the growing significance of AI-assisted monitoring and fault diagnosis frameworks in modern PV systems.

Recent advancements in DL have enabled the development of powerful temporal learning architectures such as LSTM and GRU networks for analysing time-series PV data (Talaat et al., 2026). In addition, hybrid approaches combining clustering and learning techniques have shown promising performance in improving feature representation and classification accuracy (Niazi et al., 2019). These methods integrate unsupervised and supervised learning mechanisms to better capture the complex operational behavior of PV systems under faulty and non-faulty conditions (Guessoum et al., 2024).

Limited datasets were used in the existing studies to report the classification accuracy. Furthermore, distinguishing between Normal and Mismatch Fault states is a considerable problem because of the similarity in their electrical properties. There are a few studies related to the application of hybrid approaches for enhancing the separability of features in multiclass PV fault diagnosis. In this paper, a hybrid framework of fault classification using FCM and DL models like MLP, LSTM, BiLSTM and GRU is proposed. In addition, the proposed approach considers multiclass fault classification under noisy operating conditions, making the framework more suitable for practical PV monitoring applications. The novelty of the proposed work is the fusion of fuzzy membership-based feature enhancement and the temporal DL architectures to enhance the classification of PV electrical faults. FCM algorithm provides soft membership representation, unlike the traditional preprocessing techniques, which provide better feature separability and better classification under complex operating conditions.

**Figure 1:**
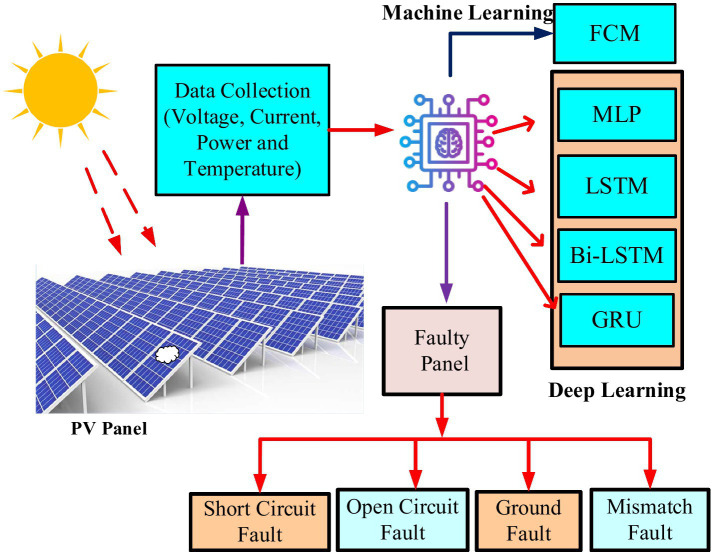
Block diagram of proposed fault classification system.

The proposed hybrid ML/DL-based PV fault classification framework is illustrated in Figure 1. The process begins with a solar PV system operating under different environmental and fault conditions. Important parameters such as voltage, current, power, irradiance, and temperature are collected through the data acquisition system and used as input features for further fault analysis and classification.

The gathered data is then analyzed through a hybrid feature extraction method that involves clustering using FCM to improve the representation of the data, discovering hidden patterns and relationships. These extracted features are, then, passed to several DL models such as MLP, LSTM, BiLSTM and GRU. These models are used for building the models by analyzing the input data, identifying the abnormal behavior in the PV system. After processing the data using models, the system determines whether the panel is experiencing normal or abnormal behavior. When abnormal behavior occurs, the system classifies the type of fault experienced by the panel into one of the four main fault categories such as OC, SC, MF and GF.

The proposed framework involves the use of intelligent methods of clustering combined with learning topologies for successful fault identification in PV panels. It uses intelligent data analysis coupled with automated classification of the results obtained to improve the efficiency and effectiveness of the systems used.

The major contribution of the proposed work as follows:

- MATLAB/Simulink was employed to analyze the impact of different faults in the PV system through voltage, current, power, and temperature variations.
- A two-stage fault diagnosis architecture is developed which combines FCM for advanced feature extraction with DL classifiers, enabling effective feature grouping and classification of faults.
- The proposed framework is evaluated using standard performance metrics such as accuracy, precision, recall, F1-score, confusion matrices, and learning curves.
- The proposed hybrid framework evaluates multiple recurrent DL models including MLP, LSTM, BiLSTM and GRU under different irradiance, temperature, and environmental conditions.

The article is outlined as follows: Mathematical modeling of PV system is explained in section 2. Sections 3 and 4 describe the different of faults and the proposed ML/DL Algorithms, respectively. Section 5 illustrates with the results of the study. The proposed work is presented in comparison with the existing works in Section 6. The conclusion of the article is presented in Section 7.

## 2 PV modeling

**Figure 2:**
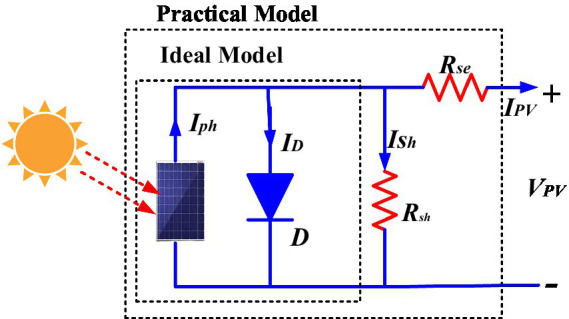
Equivalent circuit of single PV solar cell.

The equivalent circuit model is commonly used to accurately represent their electrical behavior, as illustrated in Figure 2. The most common model is the one-diode model, which is a good representation of the internal properties and functioning of solar cells. It provides an optimum computational cost while maintaining a good modeling accuracy, thus it can be used for system-level analysis and fault detection applications. Additionally, it aligns well with the standard parameters provided in commercial PV module datasheets and enables faster simulation response (Khandeparkar et al., 2025). Equation 1 describes the I-V characteristics of a PV panel with Ns based on the one-diode model and the properties of p-n semiconductors.

The I-V characteristics of a PV panel using the one-diode model is represented in Equation 1, (Khandeparkar et al., 2025).


$$I_{\mathrm{PV}}=I_{\mathrm{Ph}}-I_{D0}(\exp[\frac{q(V_{\mathrm{pv}}+I_{\mathrm{pv}}R_{\mathrm{se}})}{N_{s}\mathrm{kTa}}]-1)-\frac{V_{\mathrm{pv}}+I_{\mathrm{pv}}R_{\mathrm{se}}}{R_{\mathrm{sh}}}$$ (1)

Where, $I_{\mathrm{PV}}$ – current source, $I_{D0}$ – leakage current of the diode, q - charge $,N_{s}$ – Total cells in series, k - Boltzmann constant, T -solar cell’s temperature (K), a – ideality factor, $R_{\mathrm{se}}$ and $R_{\mathrm{sh}}$ - Series and shunt resistance, $V_{\mathrm{pv}}$ and $I_{\mathrm{pv}}$ -PV voltage and current.

In practical operating conditions, solar cells within the same PV module are typically exposed to nearly uniform irradiance, allowing them to be assumed as identical in terms of electrical characteristics and operating behavior. This assumption simplifies the modeling process by treating the PV module as a collection of identical cells connected in series and/or parallel. This means that the accurate modeling and simulation of the PV modules becomes essential when considering normal operation and failure conditions in PV systems (Noura et al., 2025).

**Figure 3:**
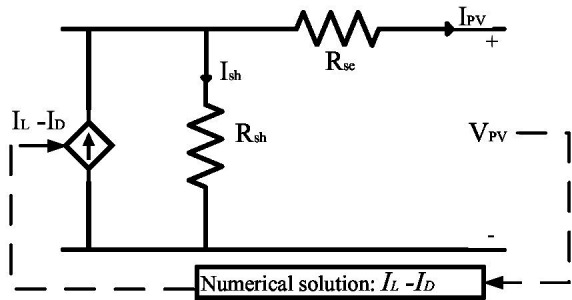
The numerical one – diode model for PV.

**Figure 4:**
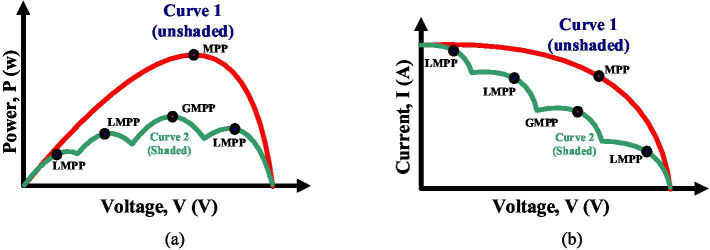
PV characteristics during shading **(a)** P-V Curve, **(b)** I-V Curve.

Bypass diodes are added in parallel with the groups of cells to avoid hotspots and minimize power losses, especially in non-uniform conditions like partial shading. The one-diode equivalent model of PV module (as shown in Figure 3) determines the following important input parameters: PV voltage, temperature, and solar irradiance. Subsequent to these inputs, the governing nonlinear equations are solved to calculate the output current and then simulated by a controlled current source in the simulation environment. This modeling can be achieved by a compromise between efficiency and accuracy, suitable for system-level analysis and fault diagnosis. Figure 4 shows the P-V and I-V characteristics of the PV panel, showing how the PV panel’s voltage and power change with current in both normal and shading fault conditions. The developed PV model was implemented and analysed under standard test conditions in the MATLAB/Simulink environment using standard PV system parameters. The PV array is composed of 20 polycrystalline PV modules in 4 series and 5 parallel, with a total power rating of about 10 kW, an open circuit voltage of 198 V and a short circuit current of 70 A.

## 3 Different types of faults in PV system

### 3.1 OC fault

Series faults in electrical and PV systems often occur because of problems like cable connection failures, faulty transmission lines, overheads, and fusing or opening of conductors or fuses in one or several phases (Roberts et al., 2001; Rahman and Srivastava, 2026). Series faults tend to occur in cases where the protective equipment, such as a circuit breaker or fuse, fails to interrupt the flow of current in all the phases. In other words, one or two phases might be open while one remains active, thus causing a series of OC faults (Zwirtes et al., 2025).

In the proposed MATLAB/Simulink model, the fault is simulated by removing one or more PV modules from the string. It significantly reduces the array current and total power. The output current under OC fault can be written ﻿in Equation 2:


$$I_{\mathrm{OCF}}=(1-\alpha_{\mathrm{OC}})I_{N}$$ (2)

Where, $I_{\mathrm{OCF}}$ - total current during OC fault, $I_{N}$ – current under healthy state, $\alpha_{\mathrm{OC}}$ *-*fault severity factor.

### 3.2 SC fault

The SC fault takes place when there is an accidental connection between two points of different potentials through a low resistance path. SC faults are some of the most serious electrical faults that take place in electrical and PV systems, since they result in a flow of large amounts of current, leading to overheating, insulation breakdowns, and even damage to system components if not rectified immediately (Alrifai et al., 2026).

In the proposed MATLAB/Simulink model, the fault is formed by providing low resistance path across the PV modules. The fault state causes a sudden rise in current, and significant drop in voltage. In addition, the output power decreases consequently. The output terminal voltage under SC fault $\left(V_{\mathrm{SCF}}\right)$ can be written ﻿in Equation 3:


$$V_{\mathrm{SCF}}=I_{\mathrm{PV}}R_{f}$$ (3)

Where, $I_{\mathrm{PV}}$ – PV current, $R_{f}$ *-*fault resistance.

### 3.3 Ground fault

Ground Fault (GF) is defined as an unintended electrical connection between an active conductor and the ground. The severity and behavior of GF conditions depend on the grounding configuration of the PV system. In low-impedance grounding systems, large fault currents may flow during a ground fault, requiring immediate isolation to ensure equipment protection and personnel safety (Karthik and Ponnambalam, 2025; Yahyaoui et al., 2025).

In the developed simulation model, the fault resistance is used to provide the leakage path between the PV conductor and the ground. This fault state reduces overall system efficiency and also creates fire risks in severe fault states. The leakage current can be written ﻿in Equation 4:


$$I_{\mathrm{GF}}=\frac{V_{\mathrm{PV}}}{R_{g}}$$ (4)

Where, $V_{\mathrm{PV}}$ -PV voltage, $R_{g}$ – ground fault resistance.

### 3.4 Mismatch fault

Mismatch Faults (MF) occur when different PV modules or cells operate under non-uniform electrical or environmental conditions, leading to unequal power generation within the PV array (Kataria et al., 2026). These faults are commonly caused by partial shading, dust accumulation, temperature variation, ageing effects, module degradation, manufacturing defects, or installation misalignment. Among these factors, partial shading is considered one of the most common causes of mismatch conditions in practical PV applications (Arif et al., 2026).

In the simulation model, the mismatch faults are induced by changing the irradiance in the selected PV modules while other modules are working under standard irradiance conditions. This fault causes a reduction in the total output power and provides multiple local maximum power points in V-P characteristics. In addition, the string current reduces due to the current-limiting effects of the shaded PV module. The mismatch power loss $\left(P_{l}\right)$ can be written ﻿in Equation 5:


$$P_{l}=P_{N}-P_{S}$$ (5)

Where, $P_{N}$ and $P_{S}$ – power under uniform irradiance and shading condition.

The fault severity levels for all faults are illustrated in Table 1. The levels were categorized as low, medium, and high based on the percentage of disconnected modules for OC fault, fault resistance for SC fault, ground fault resistance for GF, and percentage of irradiance reduction for MF.

**Table 1:** Fault Severity levels of various types of faults.

Severity Level	OC fault – Disconnected modules (%)	SC fault – Fault Resistance $(R_{f)}$	GF– ground fault resistance $\left(R_{g}\right)$	MF – Irradiance reduction (%)
Low	10–25%	10–20 Ω	>50 *Ω*	10–30%
Medium	25–50%	2–10 Ω	10–50 Ω	30–60%
High	>50%	0.1–2 Ω	<10 Ω	>60%

## 4 DL-based fault classification

ML techniques can be used to efficient and scalable learn complex relations between electrical and environmental parameters for fault classification in PV systems (Guneser et al., 2026). Unlike rule-based models, ML models can automatically discover hidden patterns from the massive amount of data, which helps to carry out accurate and real-time fault diagnosis. The type of DL algorithm is determined by the dataset size, features of the data, and the temporal dependency of the system (Haque et al., 2019).

### 4.1 MLP

MLP is a feed forward NN, which is employed for non-linear classification of fault patterns. It is made up of several hidden layers containing nonlinear activated function, which allows it to model complex interactions between the different input features.

The output of an MLP neuron is expressed ﻿in Equation 6:


$$y=f(b+\sum_{i}^{n}w_{i}x_{i})$$ (6)

Where $w_{i}$ - weights, $x_{i}$ - inputs, b - bias, and f(·) - activation function.

MLP provides fast computation and strong generalization, making it suitable for real-time PV fault classification tasks.

### 4.2 LSTM

LSTM is able to learn temporal relationships in the sequential data. It has separate memory cells and gating mechanism for storing long term memory. LSTM is an effective in PV systems where the fault patterns change over time (Sivakumar and Ramesh, 2026).

The input gate is gate is expressed in Equation 7.


$$i_{t}=\sigma (b_{i}+W_{i}[x_{t},h_{t-1}])$$ (7)

The hidden output state of LSTM is in Equation 8


$$h_{t}=o_{t}\cdot \tanh(C_{t})$$ (8)

Where $i_{t}$ - input gate, $h_{t}$ - hidden state, and $C_{t}$ - cell state.

### 4.3 Bidirectional LSTM

BiLSTM is an extension of the LSTM that can process sequences of data in a bidirectional manner. This allows the model to incorporate both historical and forecasted data from time-series PV data. Two processing modes improve the ability to detect subtle fault patterns, especially in changing environmental conditions (Balakumar et al., 2026).

### 4.4 Gated recurrent unit

**Figure 5:**
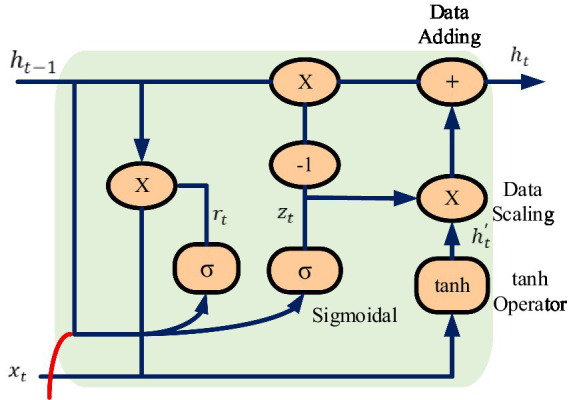
Architecture of GRU.

The architecture of GRU is shown in Figure 5. GRU is a variant of LSTM which has lower complexity without compromising performance. It provides a single update gate by merging the forget and input gates (Chen et al., 2026). The update gate is expressed ﻿in Equation 9:


$$z_{t}=\sigma (W_{z}[x_{t},h_{t-1}])$$ (9)

Where $z_{t}$ - update gate.

GRU offers quicker training and can be used in real-time PV monitoring systems that have limited computational resources.

### 4.5 Hybrid FCM-based framework

This hybrid framework allows the classifier to exploit both the clustering characteristics provided by FCM and the learning ability of DL models. FCM is used to enhance the feature representation and DL models are used for classification. FCM is first used to extract the underlying data structure and compute fuzzy membership values which are then used by the DL models to enhance feature representation and classification accuracy. The number of clusters of FCMs was chosen to be the same as the number of fault classes in the data set (C = 4), so each cluster will depict one mode of operation. The fuzzification coefficient was taken as m = 2.0, which is an appropriate value between hard and soft clustering. The clustering process was performed up to 150 iterations with convergence threshold of $10^{-5}$ . The iterative optimization terminated when the variation in cluster centers between successive iterations became smaller than the predefined threshold or when the maximum number of iterations was reached.

The preprocessed dataset can be written in Equation 10


$$X=\{x_{1},x_{2},\ldots ..x_{N}\}x_{i}є\;{\mathbb{R}}^{d}$$ (10)

Where, N - number of samples, and d -number of extracted features.

#### 4.5.1 Fuzzy C-means clustering

A preprocessing technique in order to improve the feature representation is performed by the FCM technique to cluster similar operating modes. FCM is different from hard clustering in that it assigns a membership value to each data point that can have multiple membership values to multiple clusters. This helps to make classes more separable such as Normal state and Mismatch Fault.

The objective function of FCM $(J_{m})\;$ is represented ﻿in Equation 11 (Shafiullah, 2025),


$$J_{m}=\sum_{i=1}^{k}\sum_{k=1}^{C}u_{\mathrm{ik}}^{m}{\left\|x_{i}-c_{k}\right\|}^{2}$$ (11)

Where, $u_{\mathrm{ik}}$ - membership degree of $x_{i}$ in cluster k , $c_{k}$ -cluster center, and m - fuzzification coefficient.

The cluster centers are expressed ﻿in Equation 12:


$$c_{k}=\frac{\sum_{i=1}^{N}u_{\mathrm{ik}}^{m}x_{i}}{\sum_{i=1}^{N}u_{\mathrm{ik}}^{m}}$$ (12)

The membership function $\;(u_{\mathrm{ik}})$ is expressed ﻿in Equation 13 (Zhao et al., 2018),

$$u_{\mathrm{ik}}=\frac{1}{\sum_{j=1}^{c}{\left(\frac{x_{i}-c_{k}}{x_{i}-c_{j}}\right)}^{\frac{2}{m-1}}\;}$$ (13)

FCM enhances feature extraction and improves classification performance when combined with supervised learning models.

The convergence criterion is satisfied when following conditions satisfied﻿ and it is expressed in Equation 14.


$$\|C^{\left(t+1\right)}-C^{t}\|<\varepsilon$$ (14)

Where, є=10^–5^

#### 4.5.2 Hybrid feature representation

The incorporation of FCM prior to DL classification improves the separability of PV fault patterns by grouping similar operating conditions. This enables the subsequent MLP, LSTM, BiLSTM and GRU to learn more discriminative fault characteristics, resulting in enhanced classification accuracy and robustness.

The fuzzy membership vectors $\left(U_{i}\right)$ made by FCM is written in Equation 15


$$U_{i}=[u_{i1},u_{i1},\ldots .\ldots .,u_{\mathrm{iC}}]$$ (15)

The original feature vector( $X_{i}$ ) and the corresponding membership vector are concatenated to create the enhanced feature vector $(Z_{i)}$ .


$$Z_{i}=[X_{i},U_{i}]$$ (16)

The enhanced feature vector is then given as the input to the DL classifiers.

The overall objective function of learning model is expressed in Equation 17.


$$L=\sum_{i=1}^{n}l(y_{i},{\hat{y}}_{i})+\sum_{k=1}^{K}\Omega (f_{k})$$ (17)

Where l(·) - loss function and Ω(·) - regularization.

The loss function evaluates the difference between the actual and predicted classes, which allows the model to reduce the classification errors during training. The regularization term regulates the model complexity and enhances the generation performance by decreasing overfitting. The combined objective function is used to balance classification accuracy.

### 4.6 Performance metrics

Accuracy, recall, precision and the F1 score are used to find the effectiveness of DL models for PV fault classification (Khandeparkar et al., 2025).

- Accuracy (%): It is the accuracy of the model as a whole, and is the ratio of total instances correctly classified from the total instances.
- Precision (%): This represents the percentage of positive (fault) instances correctly predicted from the total positive instances predicted by the DL model, and represents the model’s confidence level in its prediction.
- Recall (%): it is the ratio of number of actual fault cases that are properly predicted by the model to number of all total fault cases. Minimization of the number of missed faults is particular important in fault detection.
- F1-Score (%): It is a balance of recall and precision using their harmonic mean, it is appropriate in the case of class imbalance.

The mathematical formulation of these classification metrics is presented ﻿in Equations 18–21 (Kataria et al., 2026; Arif et al., 2026),


$$\text{Accuracy}=\frac{\mathrm{TP}+\mathrm{TN}\;}{\mathrm{TP}+\mathrm{TN}+\mathrm{FP}+\mathrm{FN}\;}$$ (18)

$$\text{Recall}=\frac{\mathrm{TP}}{\mathrm{TP}+\mathrm{FN}\;}$$ (19)

$$\text{Precision}=\frac{\mathrm{TP}}{\mathrm{TP}+\mathrm{FP}}$$ (20)

$$F_{1}\text{Score = 2 ×}\;\frac{\text{Precision}\times \text{Recall}\;}{\text{Precision}+\text{Recall}}$$ (21)

Where, TP – True positive, TN- True Negative, FP – False Positive, FN – Fault Negative.

The above metrics were chosen to give a comprehensive evaluation of the classification performance, where accuracy indicates the overall correctness, precision evaluates the reliability of prediction, recall indicates the ability to detect faults, and F1 score indicates a balanced measure of precision and recall.

### 4.7 Confusion matrix

The CM is an important tool for measuring the accuracy of DL models. The diagonal values of the CM are the number of instances correctly classified, and should be the largest for binary classification. In the case of multi-class classification, each row in the table represents a real class, and each column represents a predicted class with which to study the actual class-wise performance and misclassification patterns.

### 4.8 Learning curves

Accuracy curves, which are also known as learning curves, are often utilized for models that learn over time. Accuracy curves present how the training process and the effectiveness of a particular model take place. Generally, a properly trained model shows an increasing accuracy curve where the training and validation processes become more accurate with less variance. A good model usually has training and validation curves that are close together and show similar tendencies. To achieve better results and avoid overfitting, the regularization, dropout layers, and early stopping techniques, are used (Yahyaoui et al., 2025).

### 4.9 Deep learning architecture and Hyperparameter configuration

Table 2. summarizes the architectural configuration and training hyperparameters used for the proposed FCM-integrated DL models. The selection of hyperparameters was based on experiments to ensure convergence stability and balance between computing complexity. The Adam optimizer was employed in all models due to its faster convergence characteristics and adaptive learning capability. ReLU activation was used in hidden layers to reduce vanishing gradient issues, while Softmax activation was utilized in the output layer for multi-class fault classification. A larger batch size was adopted for the BiLSTM model to stabilize bidirectional sequence learning and reduce training fluctuations caused by its higher computational complexity.

**Table 2:** Deep learning architecture parameters.

Model	Architecture	Neurons	Batch size	Epochs	Learning rate	Optimizer	Activation
FCM + MLP	3 Dense Layers	128–64-32	64	40	0.001	Adam	ReLU
FCM + LSTM	LSTM + Dense	64–32	64	40	0.001	Adam	ReLU
FCM + BiLSTM	2 BiLSTM + Dense	32–32-16	128	30	0.001	Adam	ReLU
FCM + GRU	GRU + Dense	64–32	64	40	0.001	Adam	ReLU

**Figure 6:**
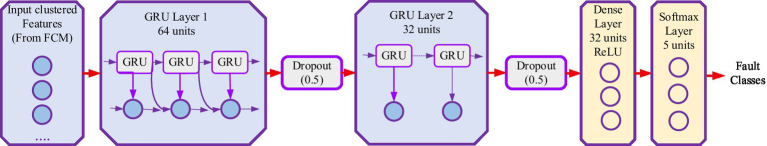
Layer-wise architecture of the FCM–GRU model.

The Layer-wise architecture of the proposed FCM–GRU model is shown in Figure 6. It employs two GRU layers, each having 64 and 32 hidden units, respectively, followed by dropout layers (0.5), a dense layer (ReLU, 32 neurons), and a Softmax output layer for the classification of PV fault categories. Features clustered by FCM provide better separability of features and better classification performance.

## 5 Results and discussion

**Figure 7:**
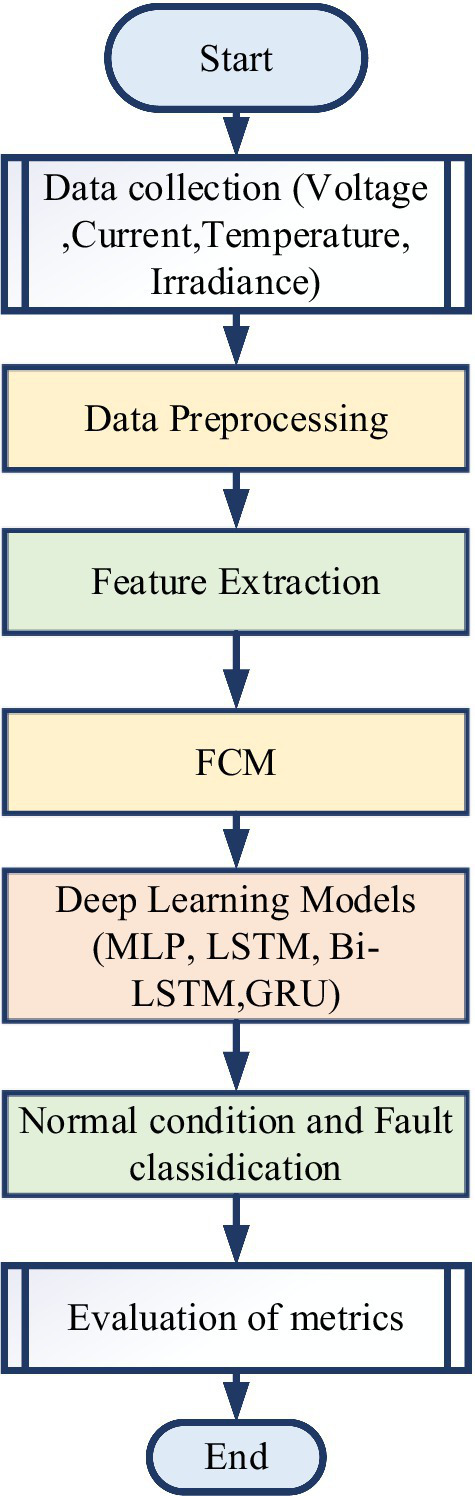
Flow process.

The flow process of the proposed PV fault classification system is shown in Figure 7. The hybrid ML models based on FCM clustering and DL methods like MLP, LSTM, BiLSTM, and GRU are used in this work.

*Data collection*: Collecting the data from the PV system is the first stage. This could include data from sensors such as V, I, and Temperature.

*Data preprocessing*: It includes data cleaning, normalization, data imputation and labeling etc.

*Model training*: The pre-processed data set is fed into the proposed hybrid FCM-based models, with 80% of the pre-processed data set dedicated to the training of the models. The models are trained to recognize patterns for different PV fault cases.

*Fault classification*: After training the model, it can classify the faults. The model processes the new input data and gives back the result showing which type of fault in the system.

*Evaluation*: The effectiveness of the models in fault detection is evaluated by analysing the metrics obtained from the confusion matrix, which include accuracy, precision, recall and F1-score.

The ranges for the benchmarking parameters are given in Table 3 according to the various fault categories. The minimum temperature limit is 25 °C, and the maximum limit is 34 °C, which is used for temperature benchmarking. These ranges are used as reference values to analyse and differentiate the PV system behavior in normal and faulty conditions. From the above observation, it can be seen that the power output is degraded in the case of MF and GF, while in the case of OC and SC, the power output is completely lost. Furthermore, the range of voltage and current differences offers good discrimination parameters, which help in the correct classification of the fault condition.

**Table 3:** Various benchmarking limits of the faults.

Sl. No	Fault Type	Power Min	Power Max	Voltage Min	Voltage Max	Current Min	Current Max
1	Normal	84	120	373	426	0.2	0.3
2	OC	0	0	371	432	0	0
3	SC	0	0	0	0	0.8	1.41
4	GF	65	96	84	112	0.7	1.44
5	MF	29	105	170	411	0.17	0.32

The dataset was collected from a 10-kW solar PV panel and comprises normal, OC, SC, GF and MF fault situations under varying irradiance and temperature levels. The input parameters and Dataset details are listed in Table 4. The input feature vectors consist of PV array voltage, current and power. These features are standardized, clustered using FCM and then used as the inputs to the proposed DL models. Prior to model training, the noisy signals were processed using a moving average filtering technique to smooth out fluctuations while preserving critical fault-related features. This preprocessing step enhances the reliability and stability of the machine learning models. To ensure a more accurate evaluation and enhanced generalization of the proposed model, a 5-fold cross-validation process was utilized.

**Table 4:** Parameters and dataset details.

Sl. No.	Parameter	Description
Input parameters		
1	Irradiance	600 W/m^2^–1,000 W/m^2^
2	Temperature	25 °C to 34 °C
3	PV array voltage	0–432 V
4	PV array current	0–1.44 A
5	PV array power rating	10kw
Dataset details		
6	Dataset Size	1,02,400 samples
7	Training/Testing splits	80% training/ 20% testing
8	Feature scaling	Standardization
9	Cross validation	5-Fold Cross Validation
10	Fault classes	normal, OC, SC, GF and MF

The electrical characteristics and behavior of each PV fault type summarized as follows:

OC: In this scenario, the voltage remains close to the open circuit voltage, and the current is 0 A. This is a definite indication of a break or disconnect in the circuit.
SC: This situation is when the voltage drops quickly to near zero volts, and the current rises quickly to an excessive voltage. Typically refers to a transmission where no load is included in the direct route.
GF: While the voltage is maintained as a steady state, a decrease in insulation resistance may be present. It indicates that the leakage current is present due to inadvertent grounding.
MF: There is a reduction in power from one or multiple panels, despite the same amount of sun exposure. The top reasons are that the shade is too dark, the modules are dirty or are old, or there is a defect in the modules.

**Figure 8:**
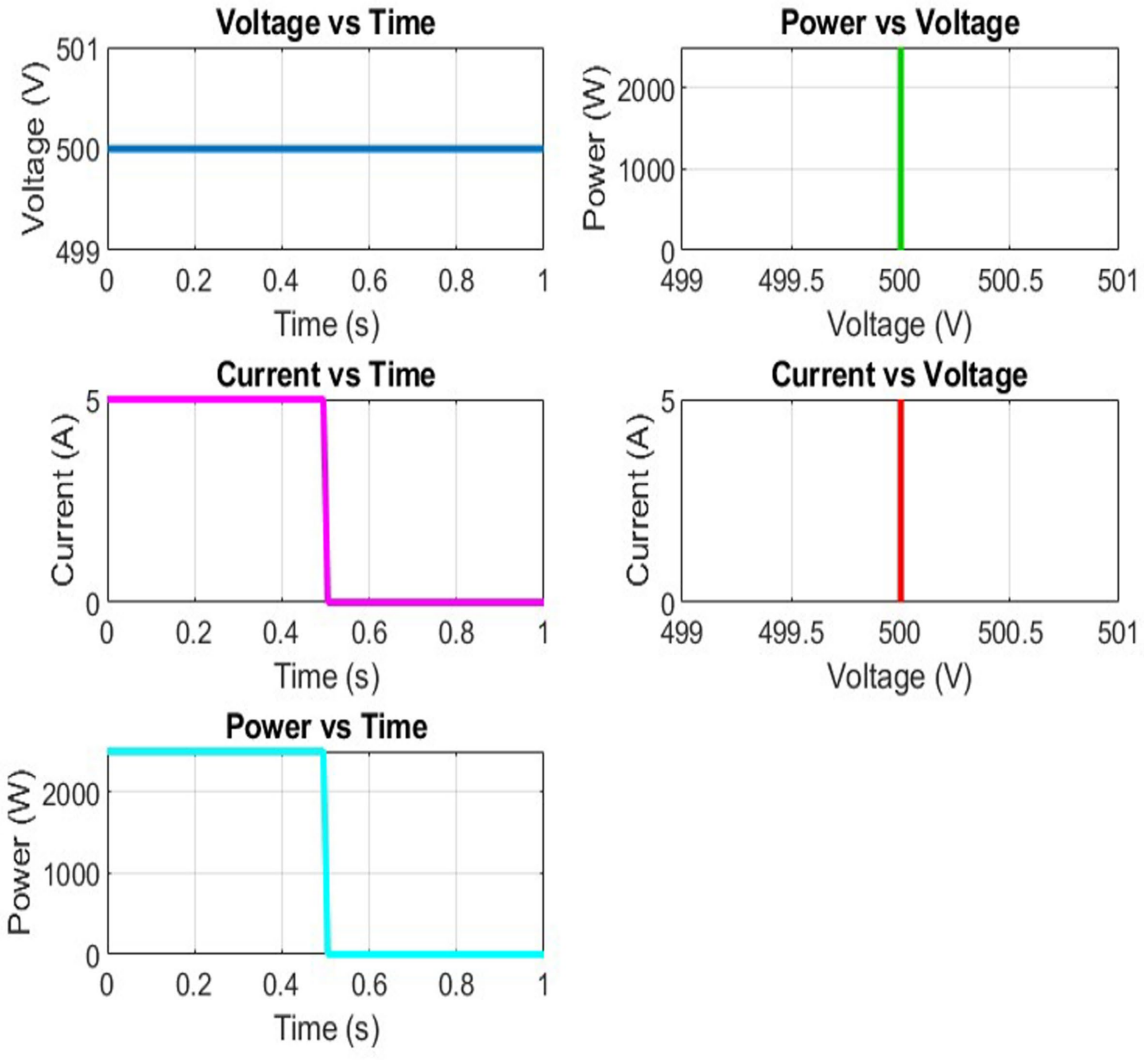
OC fault response.

The responses of Figure 8 correspond to OC fault. The OC fault was simulated in the developed MATLAB/Simulink model by disconnecting the PV string at t = 0.5 s, which also results in a zero current flow through the circuit. When operated under the OC conditions, the output current falls off rapidly to nearly zero, while the output voltage is close to the OC voltage of PV array. As a result, the generated power becomes nearly zero

- The system runs at a constant voltage of 500 V, exhibiting consistent behavior through time.
- Voltage behavior: Stays constant and hence has vertical line behavior on P-V and I-V curves.
- Current behavior: Remains consistently at 5A before falling drastically to 0A at 0.5 s.
- Power behavior: With P=VI, power remains constant at 2500 W before dropping sharply to 0 W at 0.5 s.
- Behavior of P-V and I-V curves: All changes happen at a constant voltage level, showing that the system was operating normally before the occurrence of the fault at 0.5 s.

**Figure 9:**
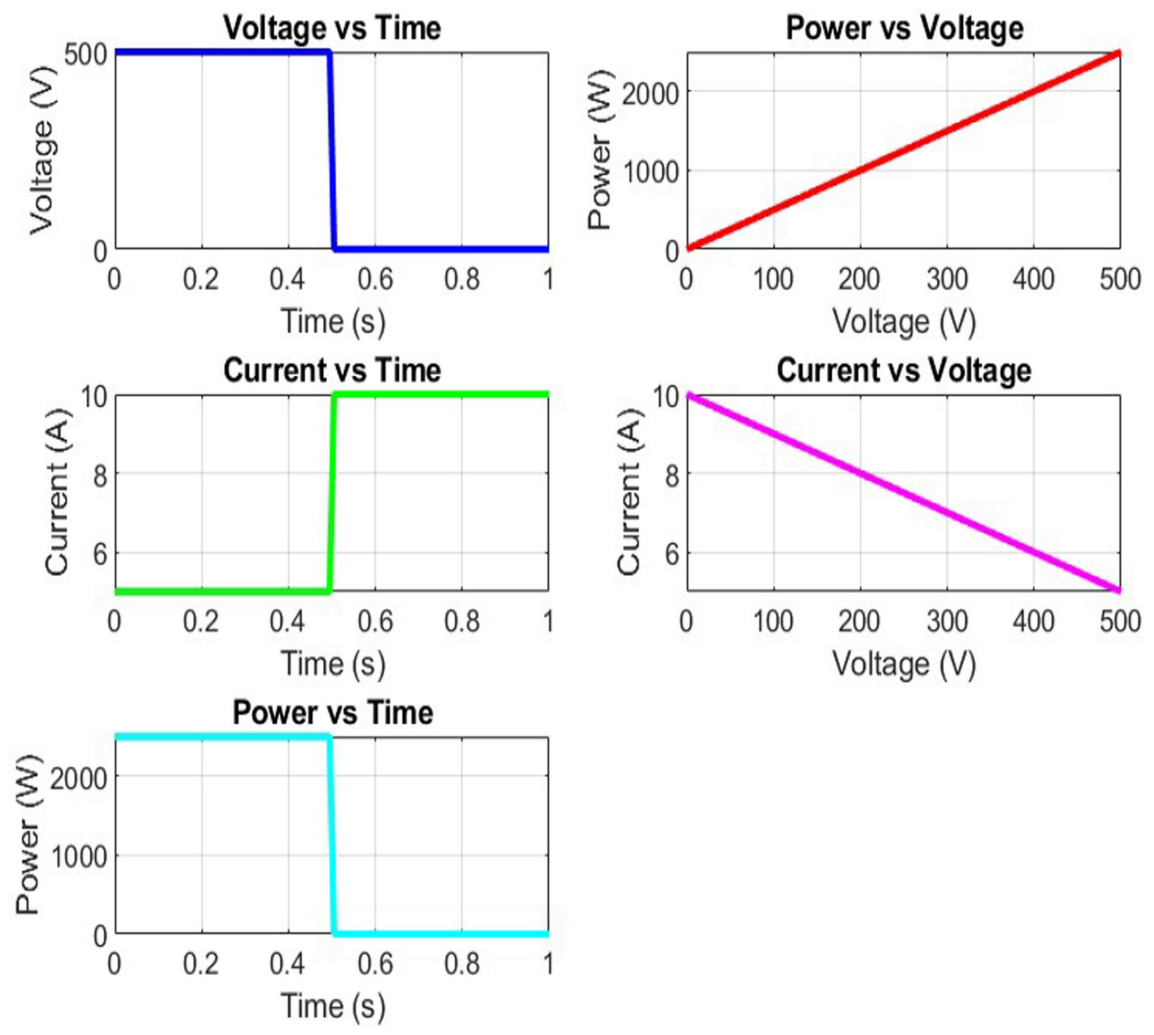
SC fault response.

Figure 9 depicts the SC fault and different curves in the MATLAB simulation. The proposed simulation model used a low resistance shunt path across the PV output terminals to simulate the SC fault at t = 0.5 s. During the fault condition, the PV voltage rapidly drops to nearly zero while the output current rises sharply beyond the normal operating range. So, the generated power collapses due to the abnormal current flow.

- There is a noticeable transition point at 0.5 s, which means there is a fault in the system.
- Voltage performance: It starts by being stable at 500 V, but suddenly falls to 0 V at 0.5 s.
- Current performance: It is consistent at 6 A until the point where it increases rapidly to 10 A at 0.5 s. Power-to-Voltage (P-V) characteristic: The result is linear, implying that power is proportional to the voltage.
- Current-to-Voltage (I-V) characteristic: There is a downward slope, implying that current is inversely proportional to the voltage.
- Power performance: It stays stable at 2000 W but then rapidly falls to 0 W at 0.5 s.

**Figure 10:**
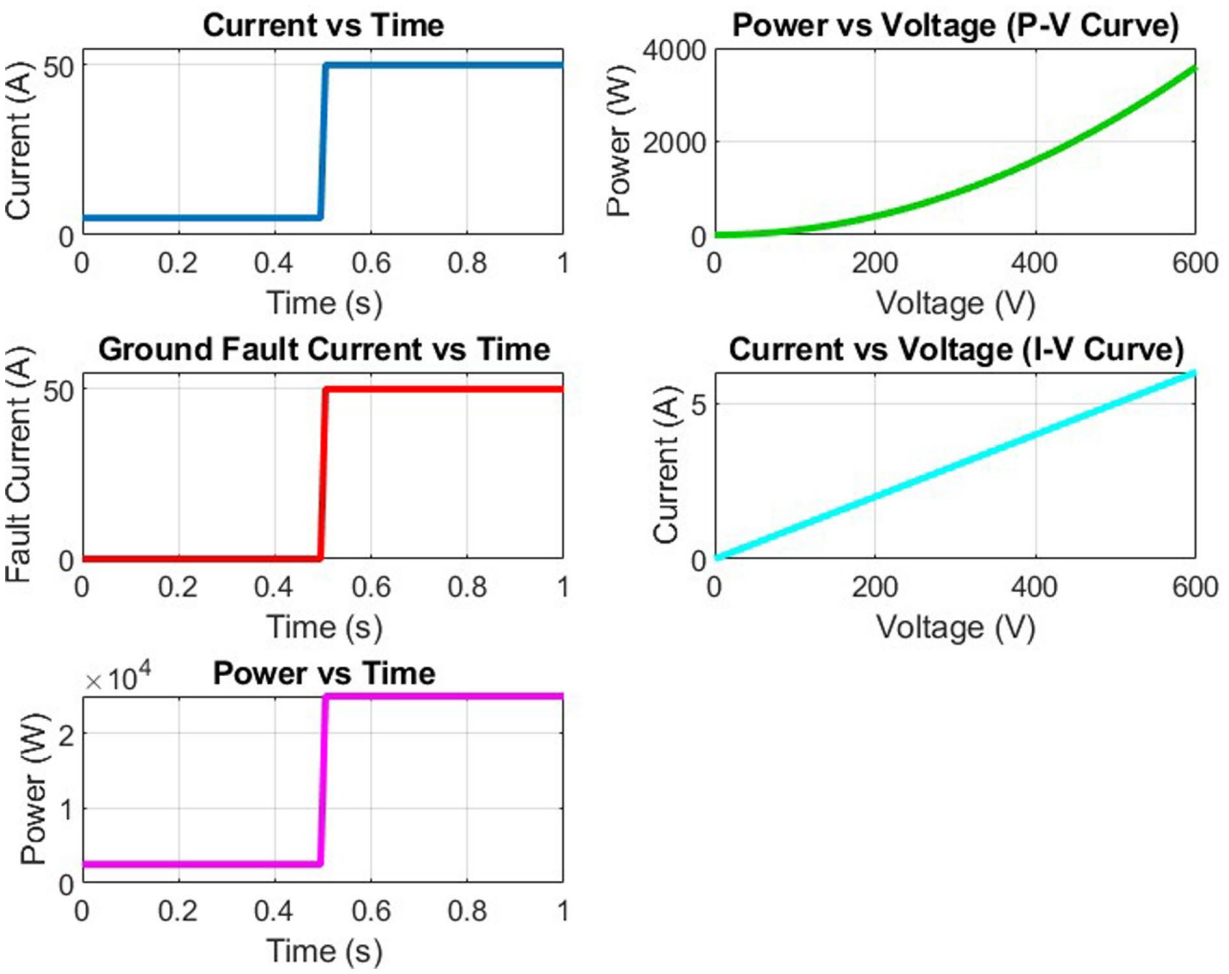
Ground fault response.

Figure 10 represent the ground fault response. The GF condition was applied to the developed PV simulation model by connecting one terminal of the PV array to ground at t = 0.5 s. When the fault occurs, the leakage current increases and the abnormal variations the voltage and power characteristics.

- An instantaneous event occurs at 0.5 s, indicating the presence of a ground fault.
- Current behavior: Initially remains at 0 A and suddenly spikes to approximately 50 A at 0.5 s.
- Ground fault current: Simultaneously increases from 0 A to around 50 A, confirming the fault occurrence.
- I–V characteristic: Shows a linear relationship, indicating proportional variation between current and voltage.
- P–V characteristic: Exhibits a non-linear (parabolic) nature, reflecting abnormal power behavior under fault conditions.
- Power behavior: Starts at a low value and abruptly rises to nearly 20,000 W at 0.5 s.
- Sharp increase in current and power is due to the sudden change in severe ground fault condition.

**Figure 11:**
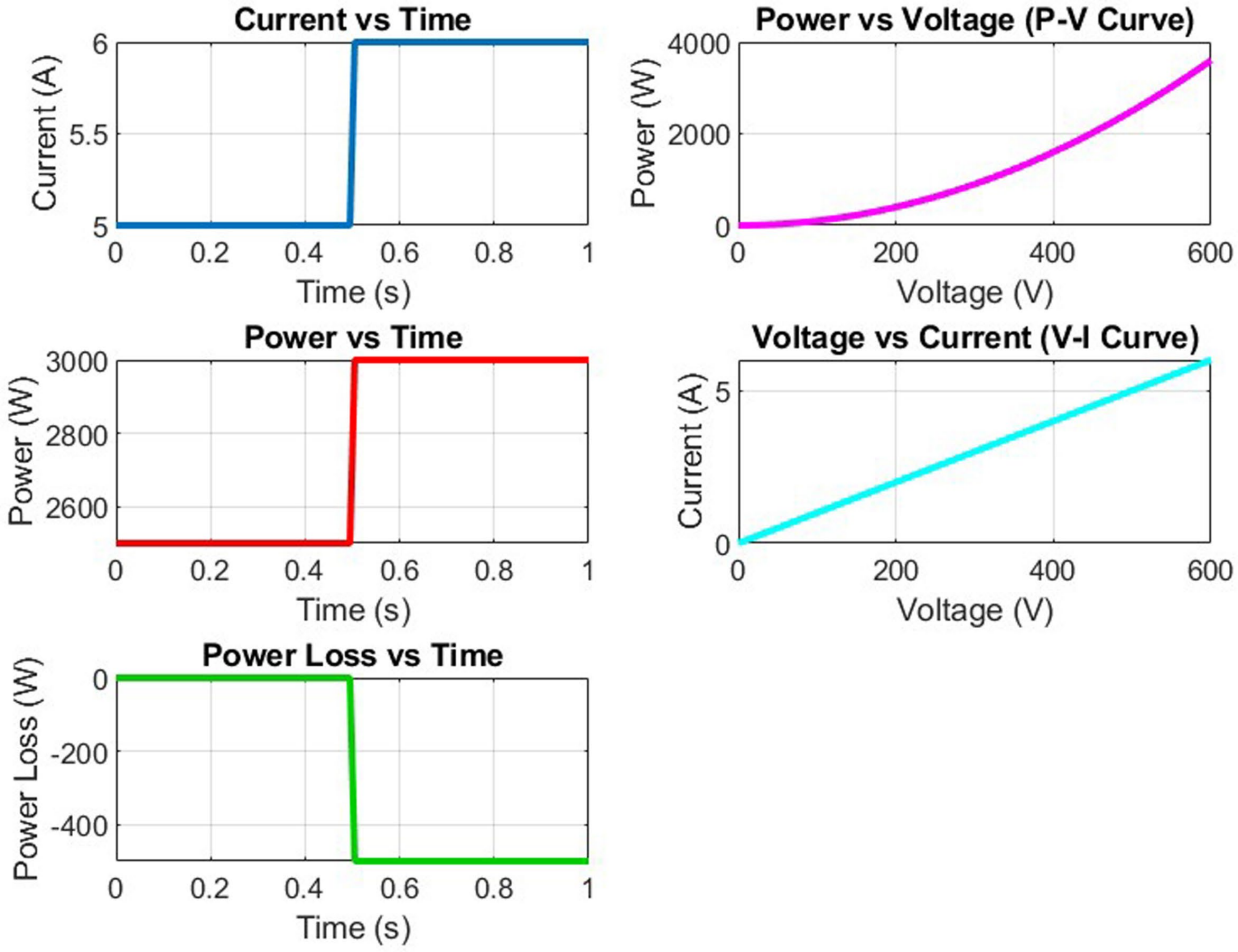
Mismatch fault response.

Figure 11 represent the mismatch fault response. In this work, the mismatch fault was generated by partially shading a portion of the PV array in the MATLAB/Simulink environment. Unlike OC and SC faults, MF does not completely interrupt the system operation; instead, it gradually decreases the output power and overall efficiency of the PV system.

- A system event occurs at t = 0.5 s, which signifies that there is a mismatch condition at fault.
- Current behavior: The current reading is slightly higher than the 5A limit, and is now approximately 6A, indicating a minor disturbance or disturbance rather than a major fault.
- V–I characteristic: It is linear, voltage varies in a proportional manner.
- P–V characteristic: It shows non-linear behavior which means power is not proportional to voltage.
- Power behavior: Steady at about 2,600 W for up to 0.5 s, followed by a rise to about 3,000 W during the event.
- Power loss: decreases from zero (pre-0.5 s) to negative (post-0.5 s) values as a result of inefficient operations due to the fault.
- These are less drastic, but still noticeable differences, that help substantiate the presence of a mismatch fault that affects the efficiency of the system, but not its failure.

**Figure 12:**
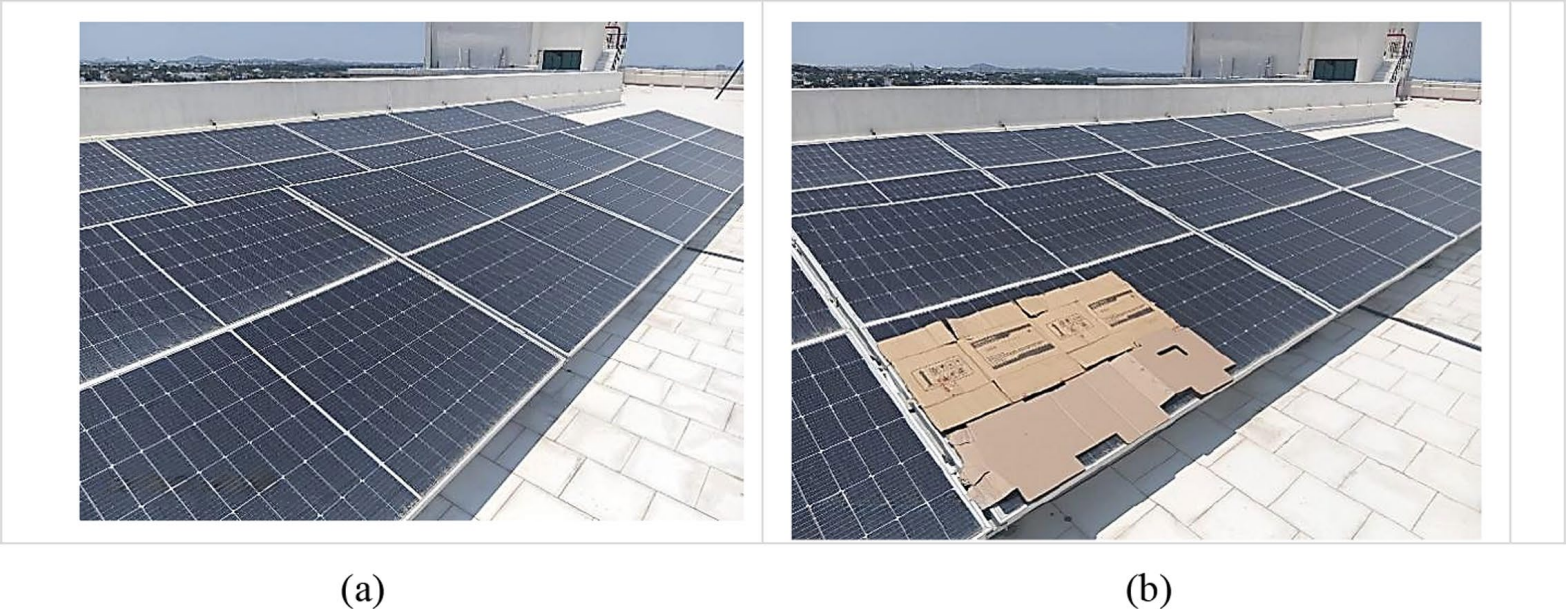
**(a)** 10 KW PV Panel. **(b)** 10 KW Shaded PV Panel.

**Figure 13:**
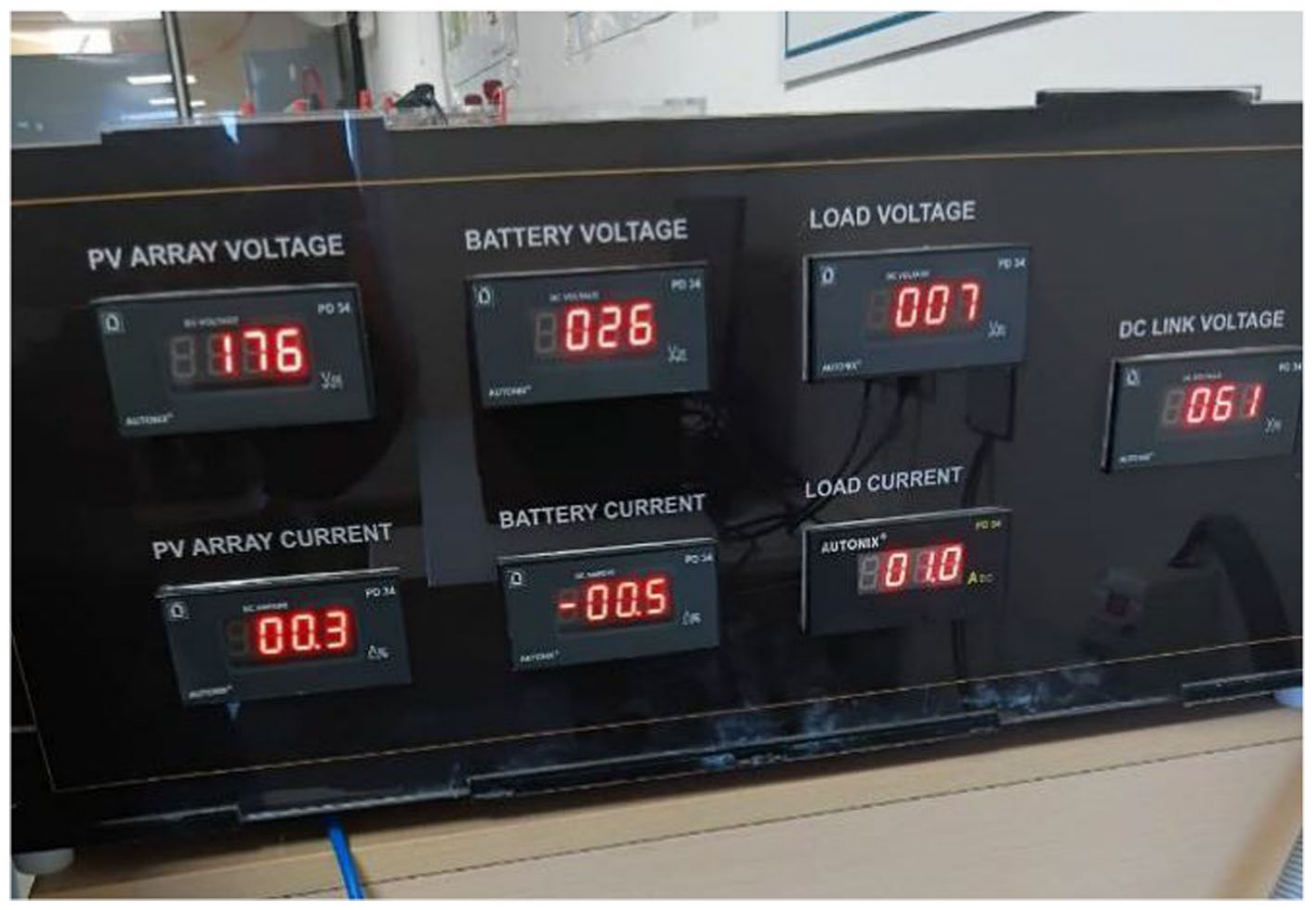
Data Visualization for PV Panel.

The setup of the 10 KW PV panel is shown in Figure 12a. We hide a part of the Solar PV panel to get the shading effect, also known as mismatch fault, as shown in Figure 12b. The data visualization for PV panel voltage and current is shown in Figure 13.

**Figure 14:**
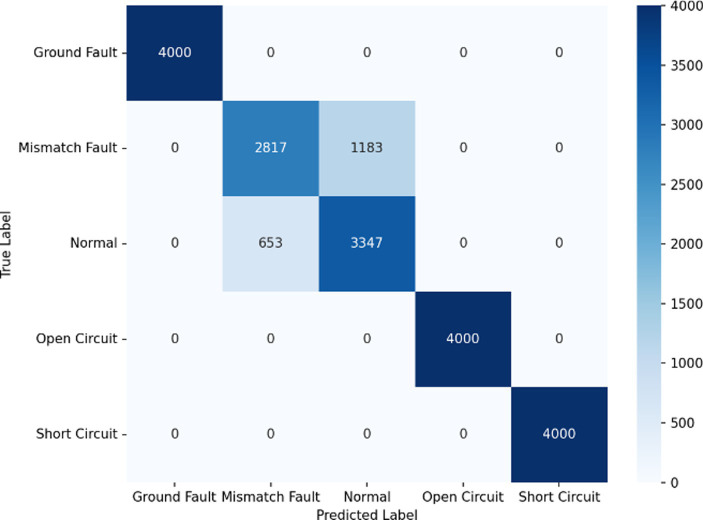
CM of FCM + MLP for all faults.

**Figure 15:**
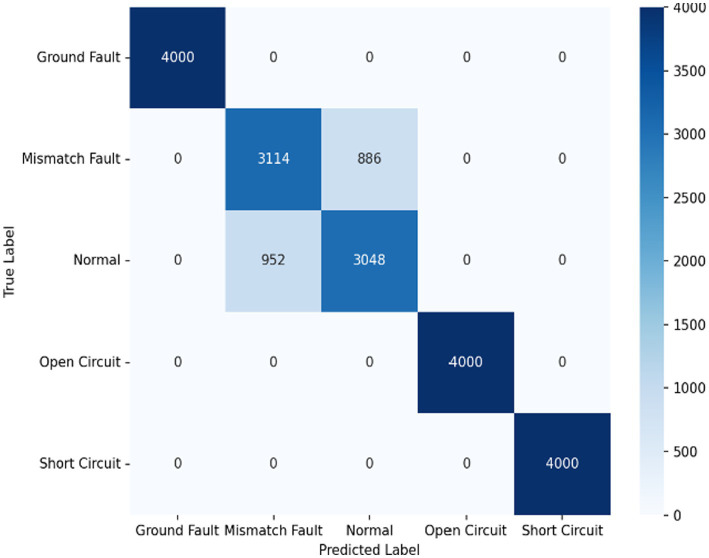
CM of FCM + LSTM for all faults.

**Figure 16:**
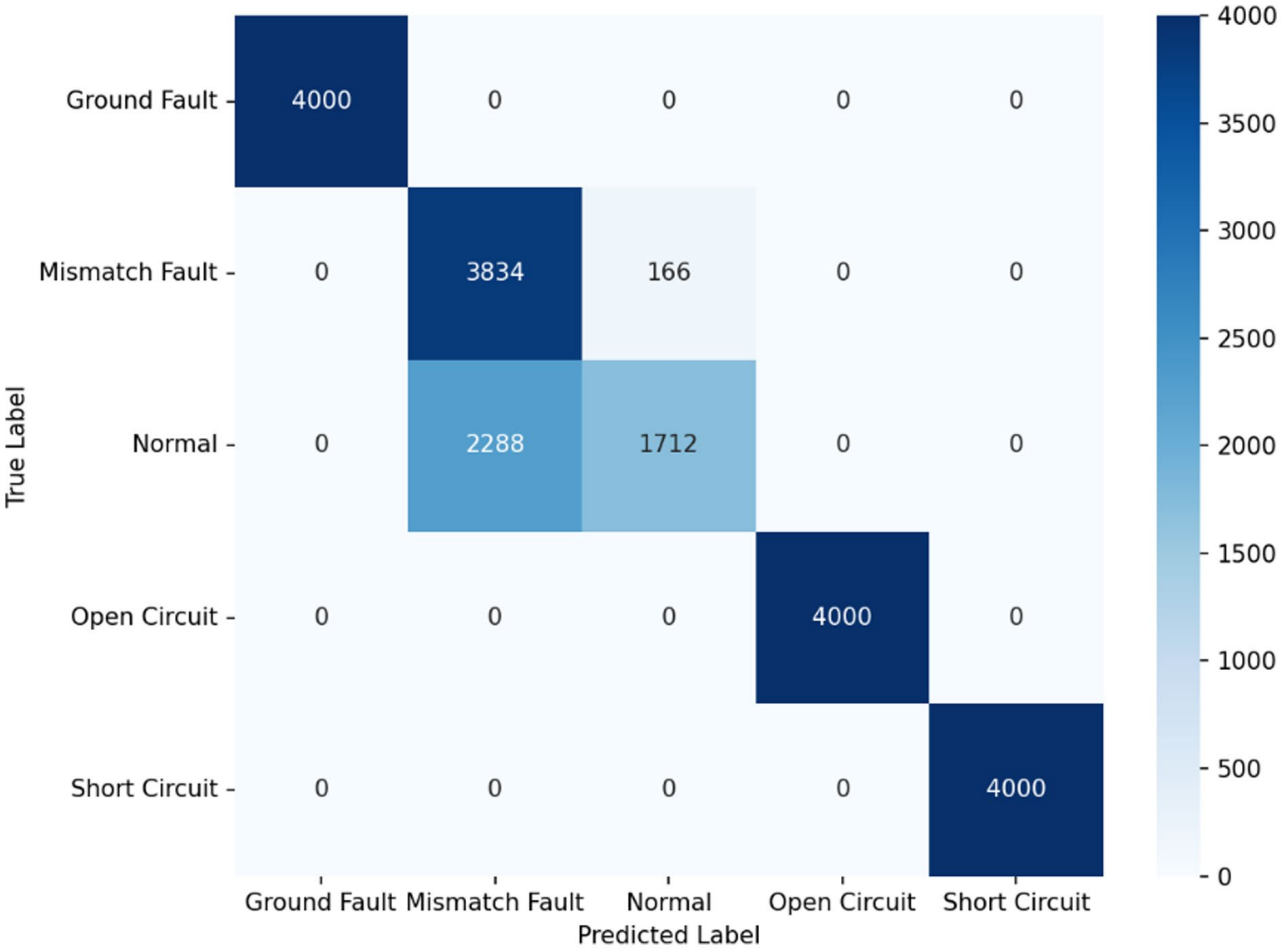
CM of FCM + Bi-LSTM for all faults.

**Figure 17:**
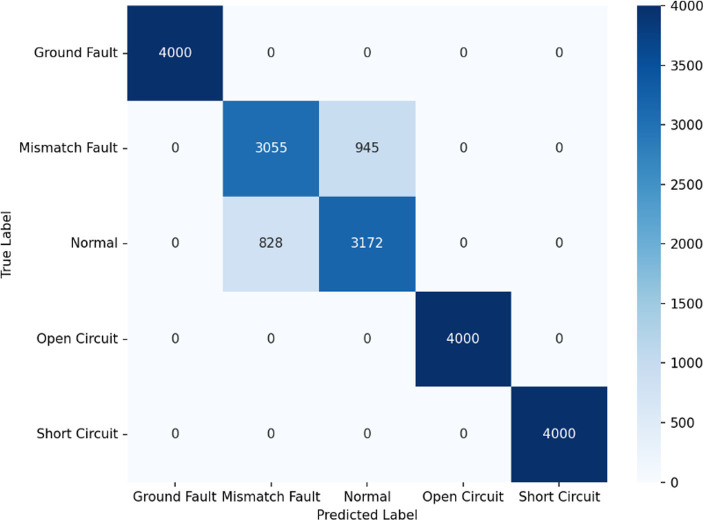
CM of FCM + GRU for all faults.

Figures 14–17 show the CM with respect to the FCM-MLP, FCM-LSTM, FCM-BiLSTM, and FCM-GRU models. From the confusion matrices, it can be seen that the four models can differentiate the major faults since all of them successfully classify normal condition, GF, OC, SC and MF fault categories perfectly. This implies that in the case of major faults, they have very distinctive features that are easily separable in their feature spaces. The only problem with misclassification occurs between normal operation conditions and mismatched conditions due to electrical similarity. The baseline FCM-MLP model demonstrates a healthy-class bias, correctly predicting 3,347 normal samples while 1,183 mismatch fault samples are wrongly classified as normals. On the contrary, the FCM-BiLSTM model presents a relatively higher sensitivity in detecting faults, where 3,842 samples are identified correctly, with only 158 samples that could not be predicted. However, this higher sensitivity presents problems with the normal condition, as there is a high probability of producing false alarms, with 2,279 samples being wrongly predicted as Mismatch Faults, while only 1,721 samples have been predicted correctly. With a balanced error rate for normal conditions and fault detection, it is evident that the FCM-LSTM model is able to predict both classes equally well, with 3,121 fault and 3,042 normal samples being predicted correctly. Nevertheless, the FCM-GRU framework works slightly better than the BiLSTM model by reducing false alarms to 838 samples while increasing the number of missed samples to 957. Consequently, with a balanced sample distribution, the GRU architecture is able to maintain the best balance between precision and recall, thereby obtaining the highest system accuracy of 91.13%.

**Figure 18:**
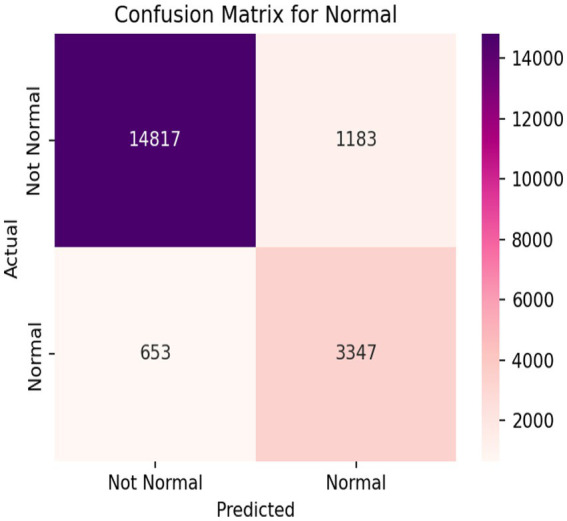
CM of FCM + MLP for normal.

**Figure 19:**
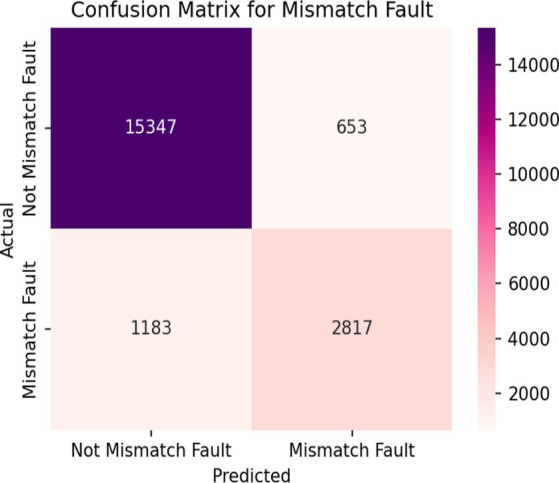
CM of FCM + MLP for MF.

**Figure 20:**
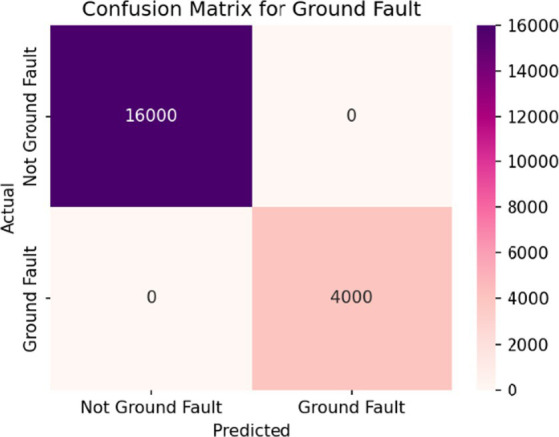
CM of FCM + MLP for GF.

**Figure 21:**
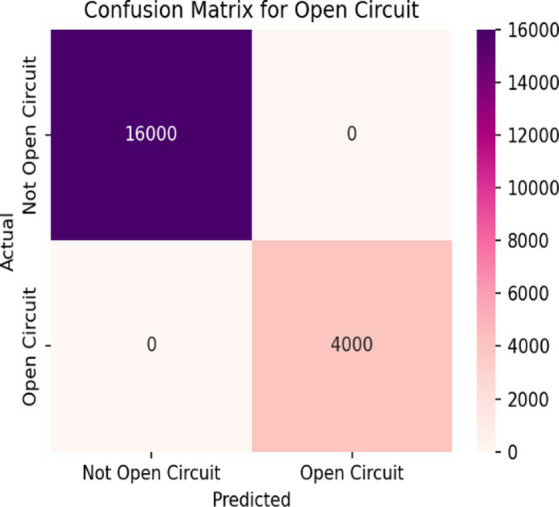
CM of FCM + MLP for OC fault.

**Figure 22:**
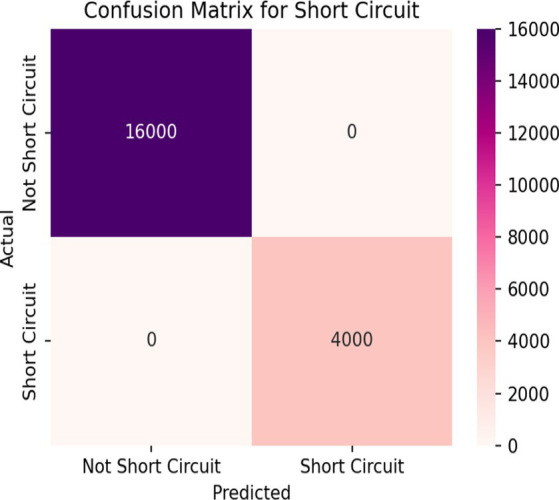
CM of FCM + MLP for SC fault.

The confusion matrices of FCM + MLP for various operational modes are provided in Figures 18–22. The proposed FCM + MLP model is able to obtain perfect classification performance for OC, SC, and GF. It proves the high capability in identifying electrical faults. Nevertheless, the proposed FCM + MLP model suffers from some misclassification of samples between the Normal condition and the MF condition, which suggests that some samples from each class are mistakenly identified as the other. In comparison to the LSTM model, the MLP model identifies the normal condition more accurately, although the mismatch fault conditions suffer a slight reduction in performance. It shows that even though the MLP model can learn temporal features, it may be biased toward normal operation.

**Figure 23:**
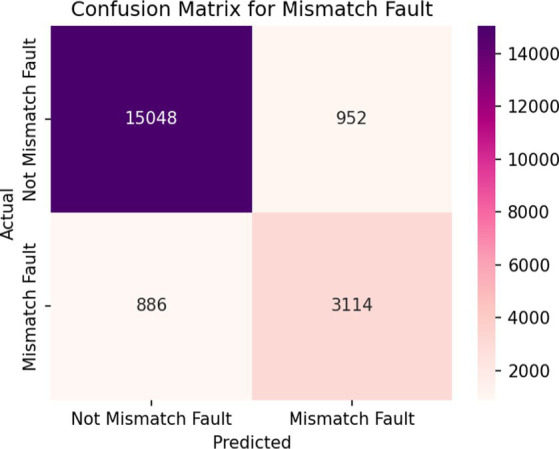
CM of FCM + LSTM for MF.

**Figure 24:**
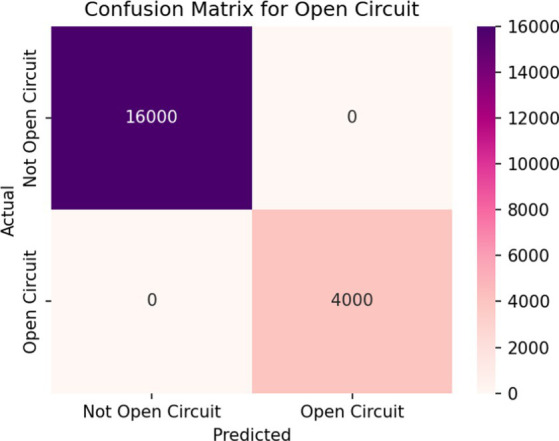
CM of FCM + LSTM for OC fault.

**Figure 25:**
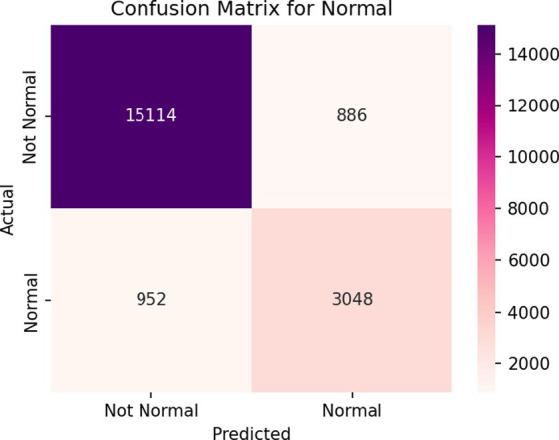
CM of FCM + LSTM for normal.

**Figure 26:**
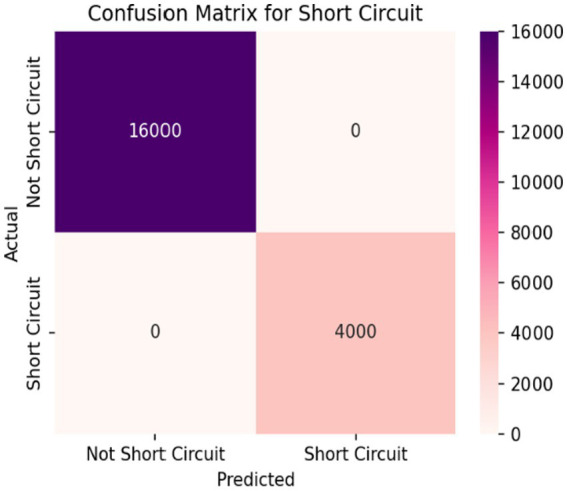
CM of FCM + LSTM for SC fault.

**Figure 27:**
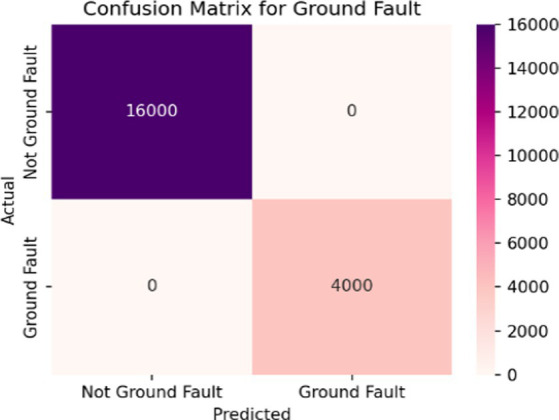
CM of FCM + LSTM for GF.

Figures 23–27 illustrate the one-vs-all confusion matrices derived from the proposed FCM + LSTM algorithm for various fault states. In terms of fault diagnosis, the algorithm demonstrates excellent performance in recognizing all electrical faults, including OC, SC, and GF. In some cases, the algorithm is capable of misclassification when Normal and MF faults are considered. However, the number of such instances is small, with examples of samples being swapped between the two fault states. These results suggest that, while the algorithm performs well in detecting the temporal features of severe faults, it struggles to differentiate the differences in more closely related operating modes.

**Figure 28:**
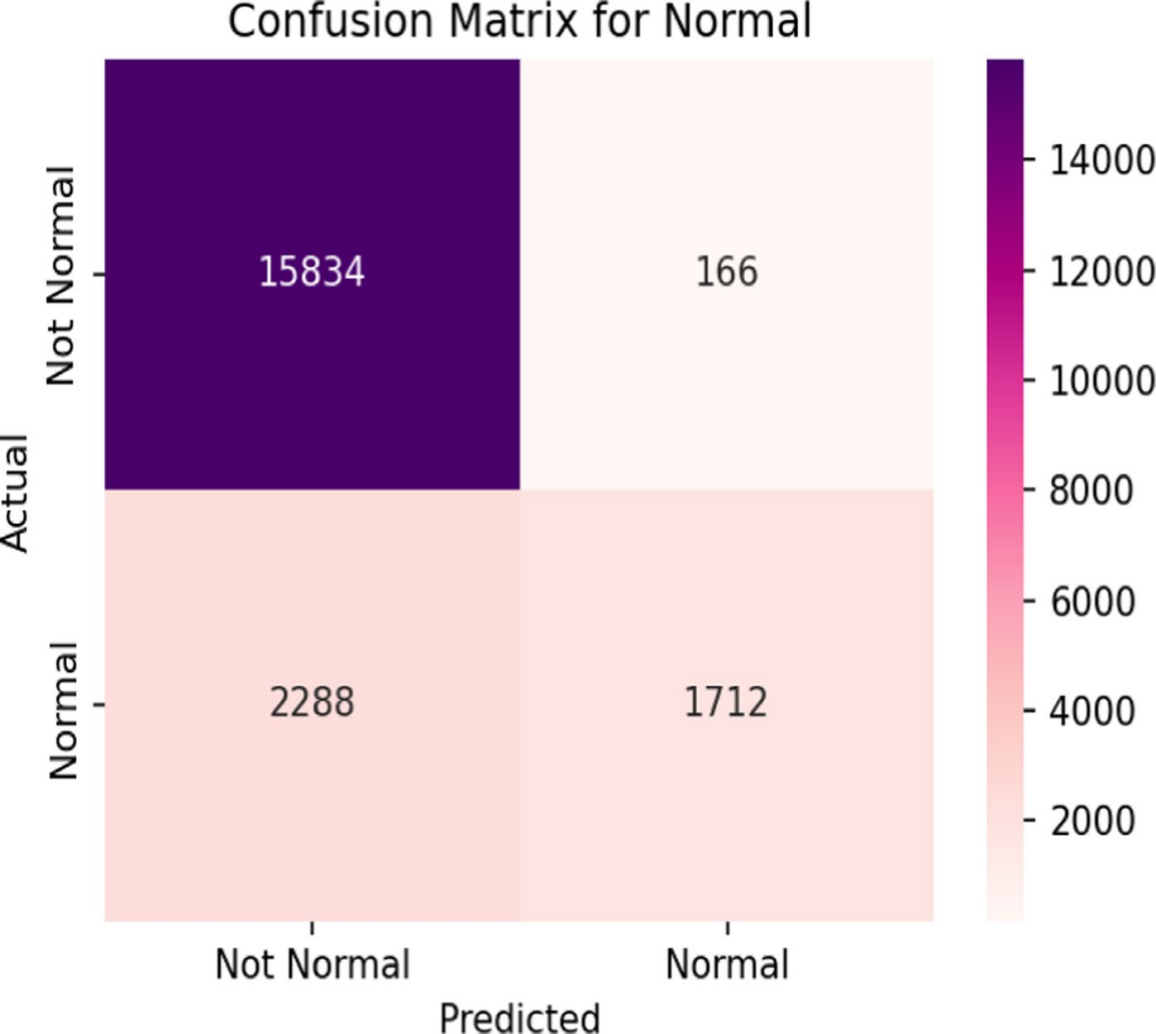
CM of FCM + BiLSTM for normal.

**Figure 29:**
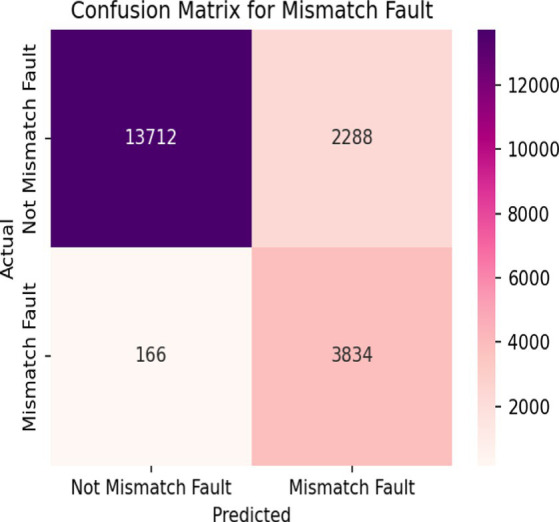
CM of FCM + BiLSTM for MF.

**Figure 30:**
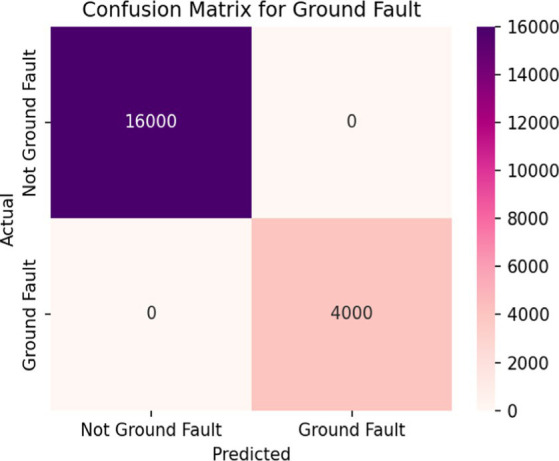
CM of FCM + BiLSTM for GF.

**Figure 31:**
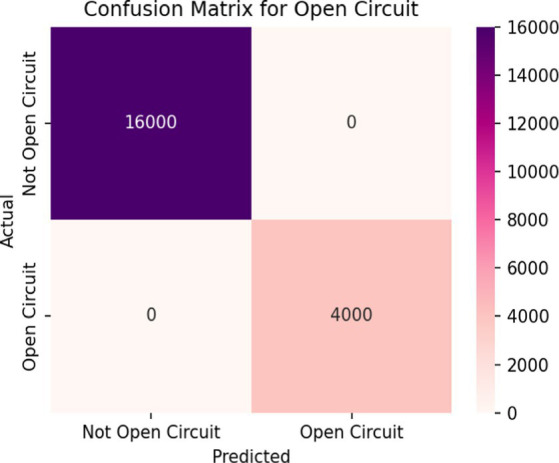
CM of FCM + BiLSTM for OC fault.

**Figure 32:**
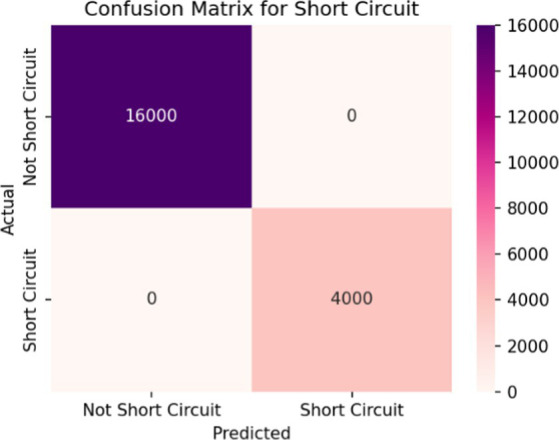
CM of FCM + BiLSTM for SC fault.

The confusion matrices for the one-vs-all classifier using the proposed FCM + BiLSTM model under various operating conditions are shown in Figures 28–32. From the plots, almost all the data points are accurately classified by the model, based on the strong diagonal values. The model perfectly classifies the GF, OC, and SC faults, with no FP or FN recorded, proving its capability in classifying critical faults. There is, however, a major misclassification of Normal vs. MF operating conditions, where there is an abundance of FP, suggesting that the model classifies more data points as faults than normal. In other words, while the FCM + BiLSTM model has extremely high recall in detecting MF, it has a low sensitivity for normal operating conditions.

**Figure 33:**
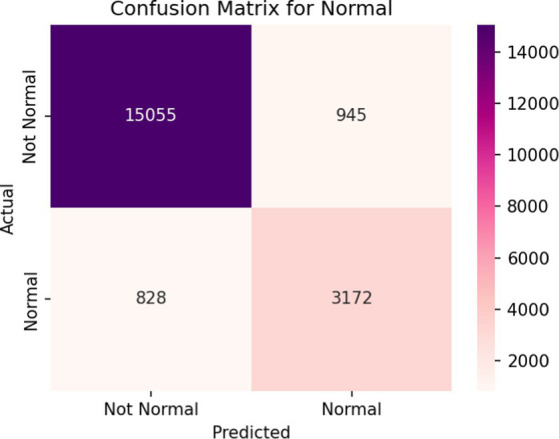
FCM + GRU for normal.

**Figure 34:**
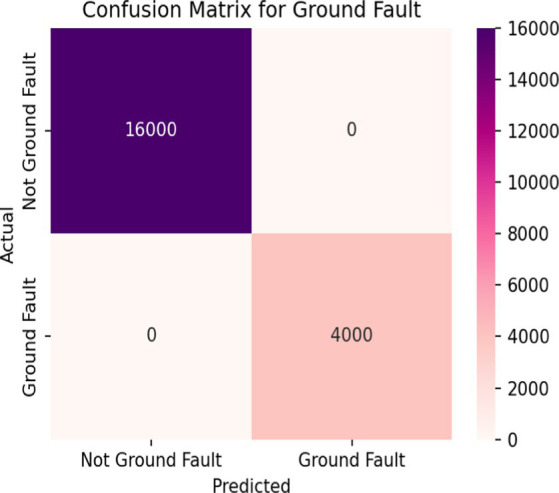
FCM + GRU for GF.

**Figure 35:**
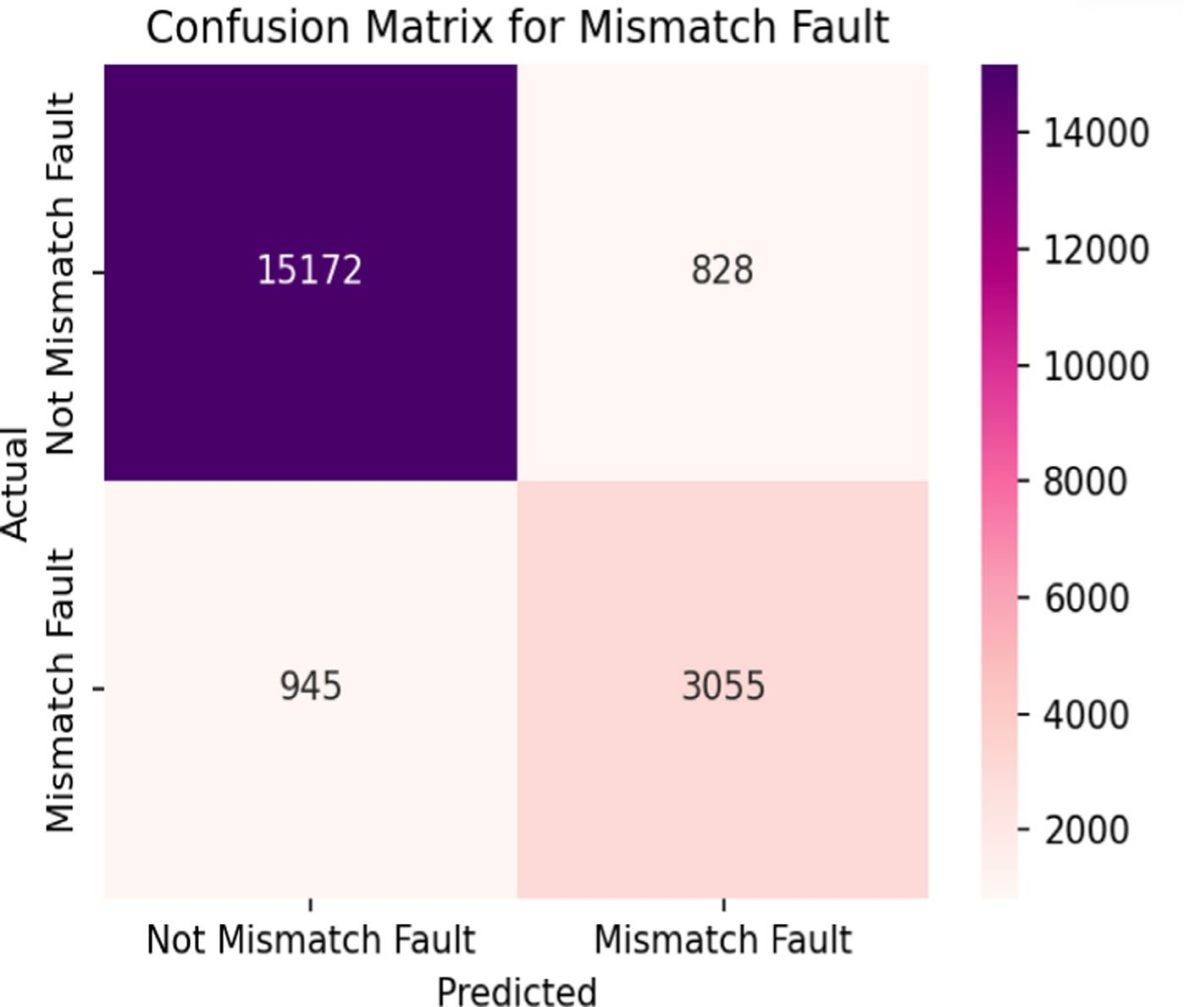
FCM + GRU for MF.

**Figure 36:**
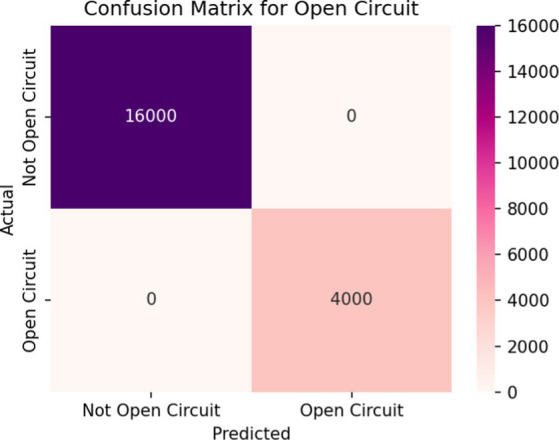
FCM + GRU for OC fault.

**Figure 37:**
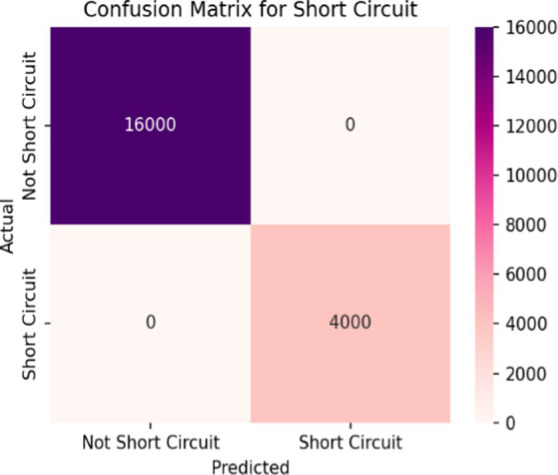
FCM + GRU for SC fault.

Confusion matrices for the one-vs-all scenario for the proposed model (FCM + GRU) for various operating conditions are illustrated in Figures 33–37. Based on the confusion matrices provided, it is evident that the samples have been mostly classified since there are high values on the diagonal. The model perfectly classifies all instances for the cases of GF, OC and SC faults. There were instances whereby samples between Normal and MF conditions were misclassified as these two classes exchanged samples between themselves. As compared to the recurrent models, the GRU model performs well in terms of being able to recognize more instances of normal operating conditions while recognizing fewer instances of Mismatch Faults.

**Figure 38:**
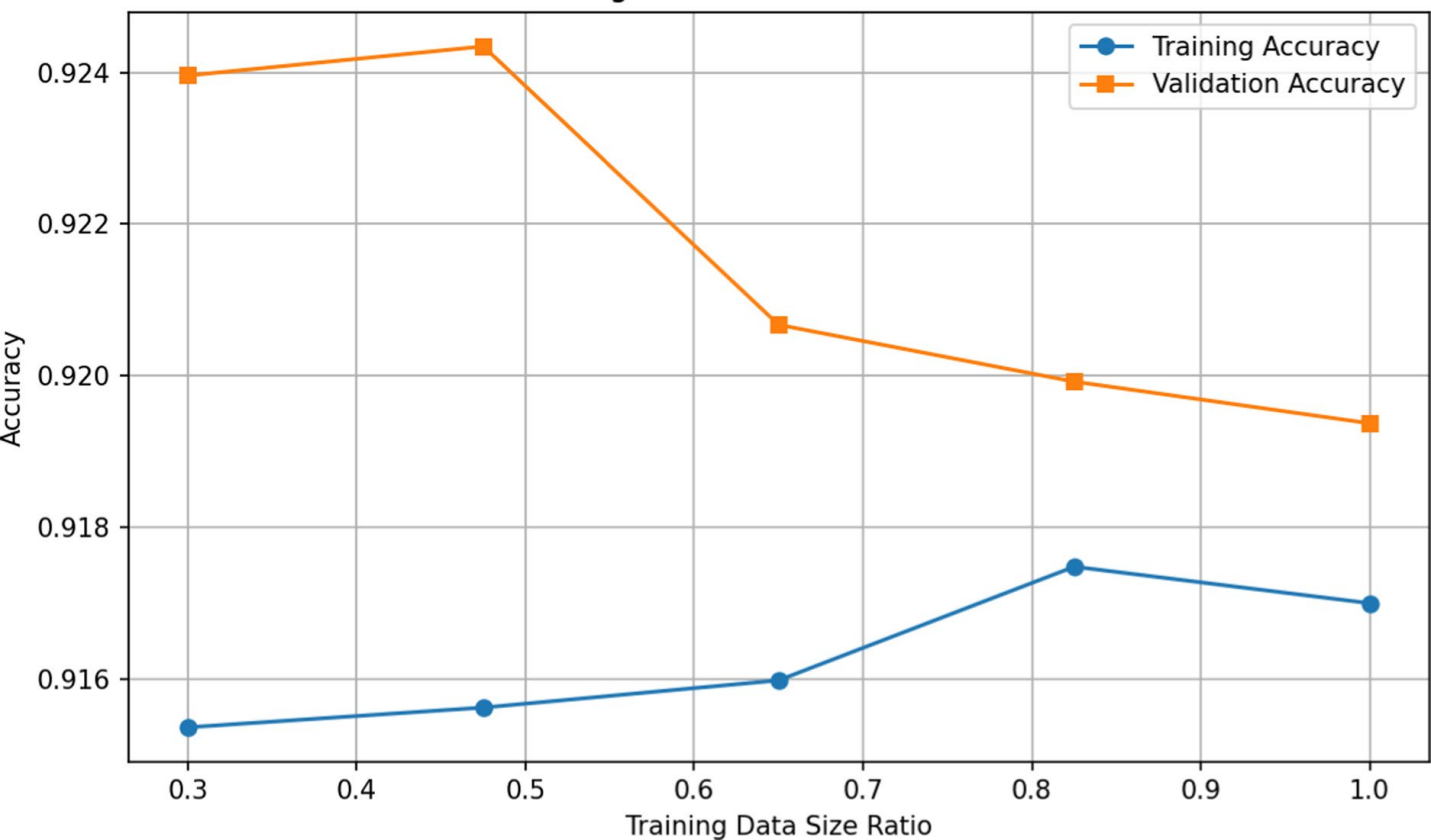
MLP learning curve.

**Figure 39:**
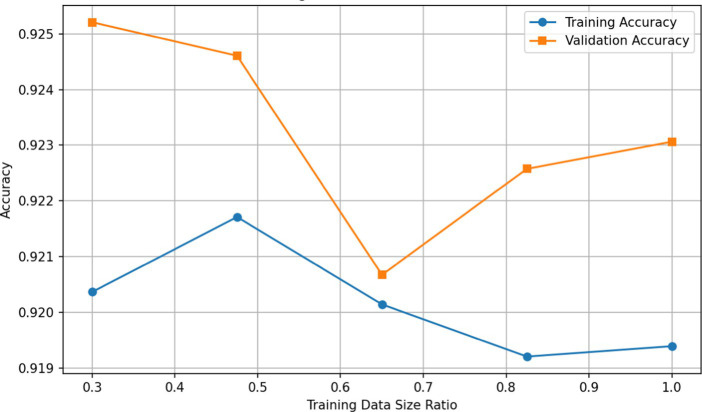
LSTM learning curve.

**Figure 40:**
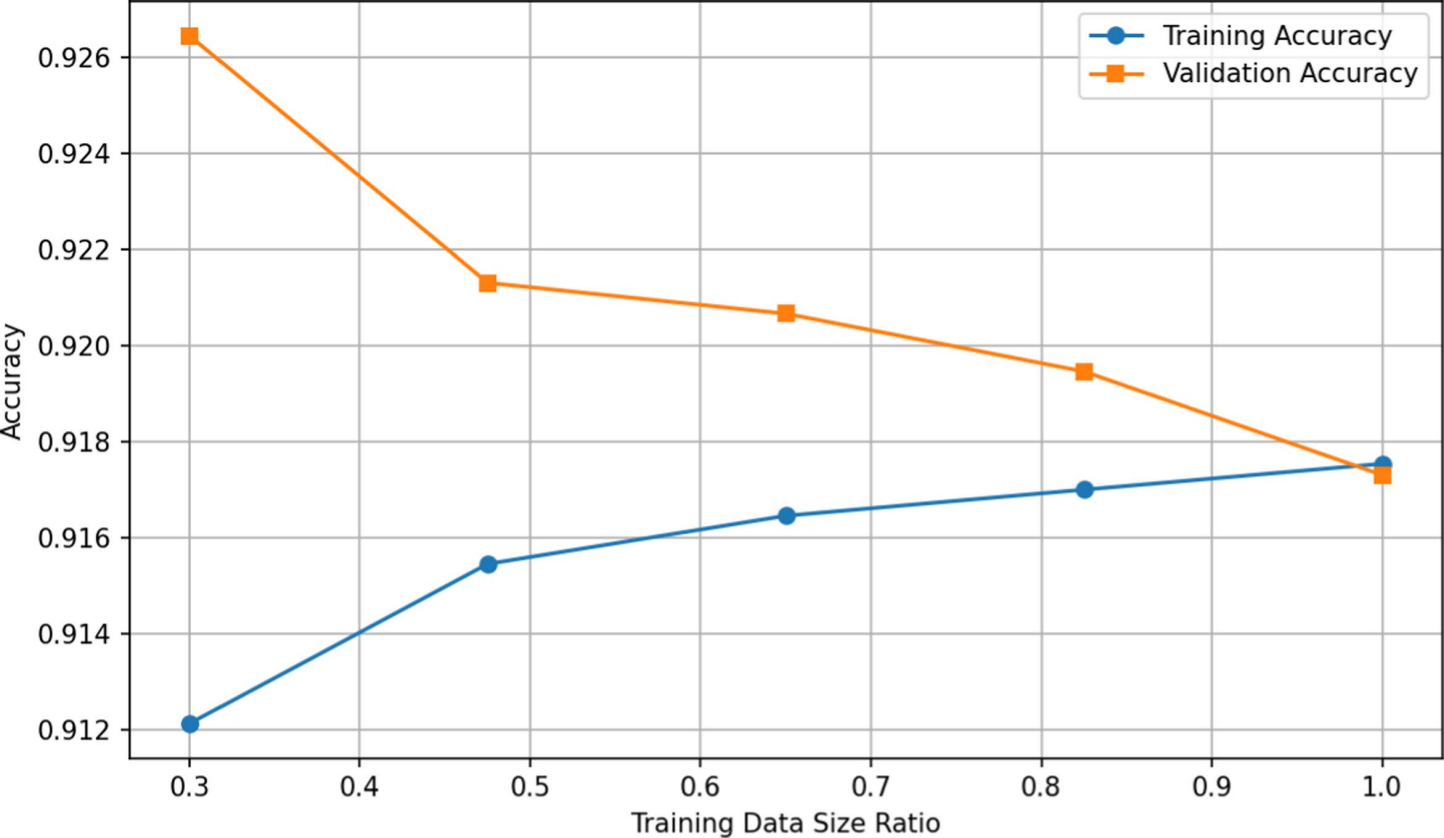
BiLSTM learning curve.

**Figure 41:**
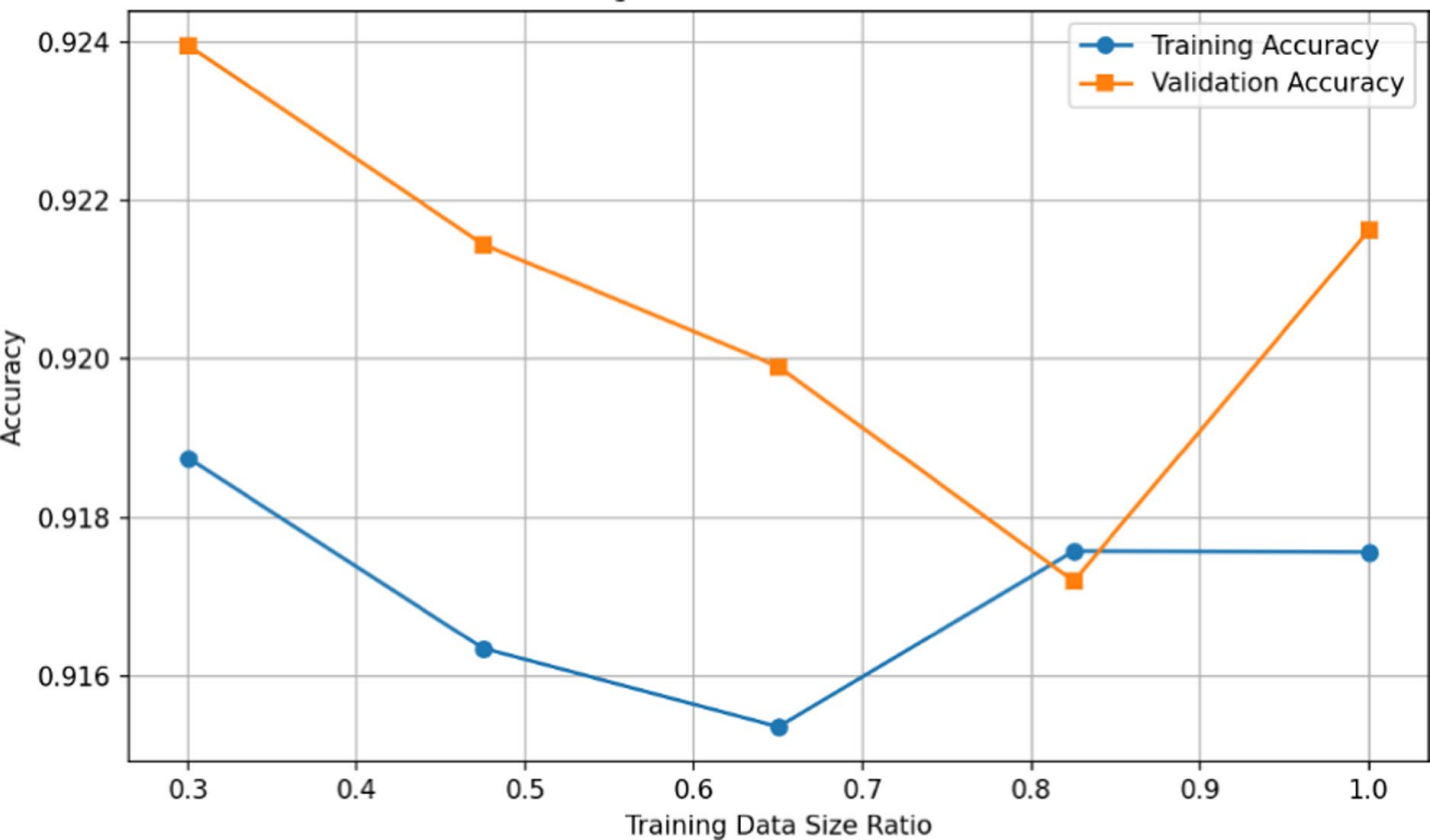
GRU learning curve.

The plots shown in Figures 38–41 are the learning curves for the FCM + MLP, FCM + LSTM, FCM + BiLSTM, and FCM + GRU models, respectively, which depict how the training and validation accuracies change as the number of data used for training increases. For LSTM and MLP models, there is stability in the training and validation accuracies; the former being just lower than the latter in most cases, thus demonstrating the consistency of the learning and its generalizability. However, for BiLSTM, the convergence of both the training and validation accuracies at 91.7% implies low variance and no overfitting. In the case of the GRU model, training and validation accuracy have significant fluctuations, showing their inconsistency and sensitivity to data variations. The brief decrease in the validation accuracy below the training one depicts some degree of overfitting that eventually gets recovered as the amount of data grows. Overall, all four models exhibit similar levels of convergence at nearly identical accuracy rates (91.7–92.3%).

### 5.1 Performance analysis

Table 5 shows the comparison of different hybrid FCM models in terms of fault classification. Multiple models analysis shows that all models perform excellently and have a very high accuracy rate, which is between 87.73 and 91.13%. Particularly, the FCM + GRU model achieves the best performance among all other models by obtaining the highest accuracy rate of 91.13% with the highest precision, recall, and F1-score rates. The superior performance of the FCM + GRU model can be attributed to the capability of FCM to improve the feature separability and the efficient gated memory mechanism of GRU, which efficiently captures the fault patterns while maintaining the lower computational complexity compared with LSTM and BiLSTM. This reduced complexity minimizes overfitting and improves convergence stability, resulting in balanced precision, recall, and F1-score performance. The performance of the FCM + LSTM and FCM + MLP models is almost equal and obtains a classification accuracy rate close to 91%. Meanwhile, the FCM + BiLSTM model has low performance of 87.73%, and the reason for this is that the model fails to classify between MF and Normal conditions.

**Table 5:** Comparison of different DL models.

DL algorithms	Faults	Precision	Recall	F1-score	Accuracy (%)
FCM + MLP	GF	1.00	1.00	1.00	90.82
	MF	0.81	0.70	0.75	
	Normal	0.74	0.84	0.78	
	OC	1.00	1.00	1.00	
	SC	1.00	1.00	1.00	
FCM + LSTM	GF	1.00	1.00	1.00	90.81
	MF	0.77	0.78	0.77	
	Normal	0.77	0.76	0.77	
	OC	1.00	1.00	1.00	
	SC	1.00	1.00	1.00	
FCM + BiLSTM	GF	1.00	1.00	1.00	87.73
	MF	0.63	0.96	0.76	
	Normal	0.91	0.43	0.58	
	OC	1.00	1.00	1.00	
	SC	1.00	1.00	1.00	
FCM + GRU	GF	1.00	1.00	1.00	91.13
	MF	0.79	0.76	0.78	
	Normal	0.77	0.79	0.78	
	OC	1.00	1.00	1.00	
	GF	1.00	1.00	1.00	
	SC	1.00	1.00	1.00	

The training time and computational complexity are shown in Table 6, which shows that FCM + MLP achieves the lowest training time (~1.5–2.5 s) with moderate complexity, making it suitable for fast implementations, though it lacks the ability to capture temporal dependencies. In contrast, sequence-based models such as FCM + LSTM and FCM + GRU require higher training time due to their sequential processing, but offer improved learning of time-dependent patterns. In particular, FCM + GRU ensures a better compromise between efficiency and performance, as it takes only slightly more time for training, approximately 3.5–5.0 s, compared to LSTM, and is more appropriate for near real-time or real-time applications, which are characterized by complex and sophisticated tasks. However, FCM + BiLSTM has the longest training time (~6.5–8.0 s) and the highest complexity because of the bidirectional processing of the system. The reduced computational complexity and lower parameter count of the GRU architecture make the proposed FCM + GRU framework suitable for real-time photovoltaic monitoring applications. Compared with BiLSTM and LSTM models, GRU achieves faster convergence and lower inference complexity while maintaining strong temporal learning capability.

**Table 6:** Comparison of model training time and complexity.

Model	Training Time (s)	Complexity
FCM + MLP	~1.5–2.5	Medium (O(n·d))
FCM + LSTM	~4.5–6.0	High (O(n·t·d))
FCM + BiLSTM	~6.5–8.0	Very High (O(2·n·t·d))
FCM + GRU	~3.0–5.5	High (O(n·t·d))

**Figure 42:**
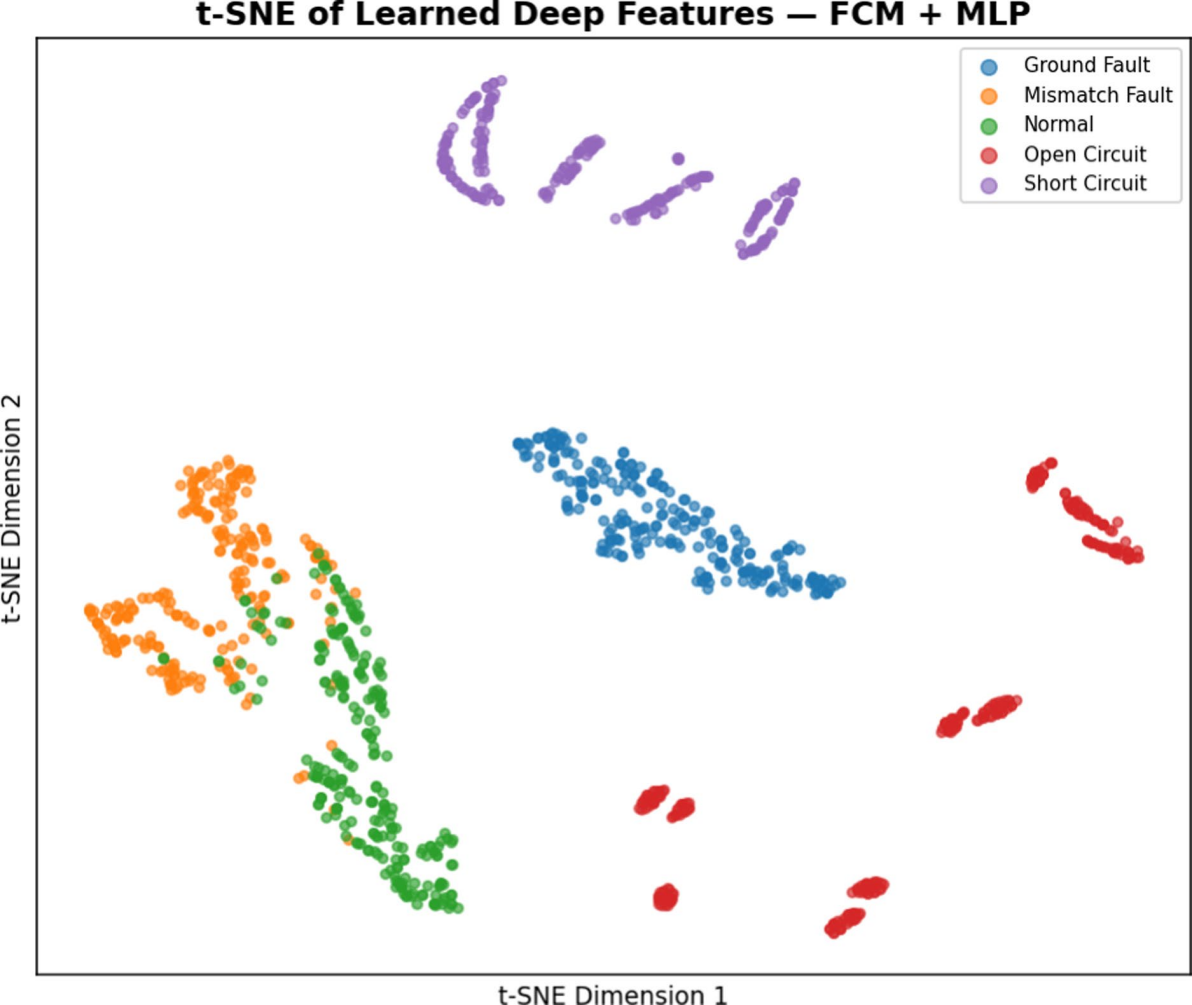
t-SNE visualization of PV fault classes for FCM + MLP.

**Figure 43:**
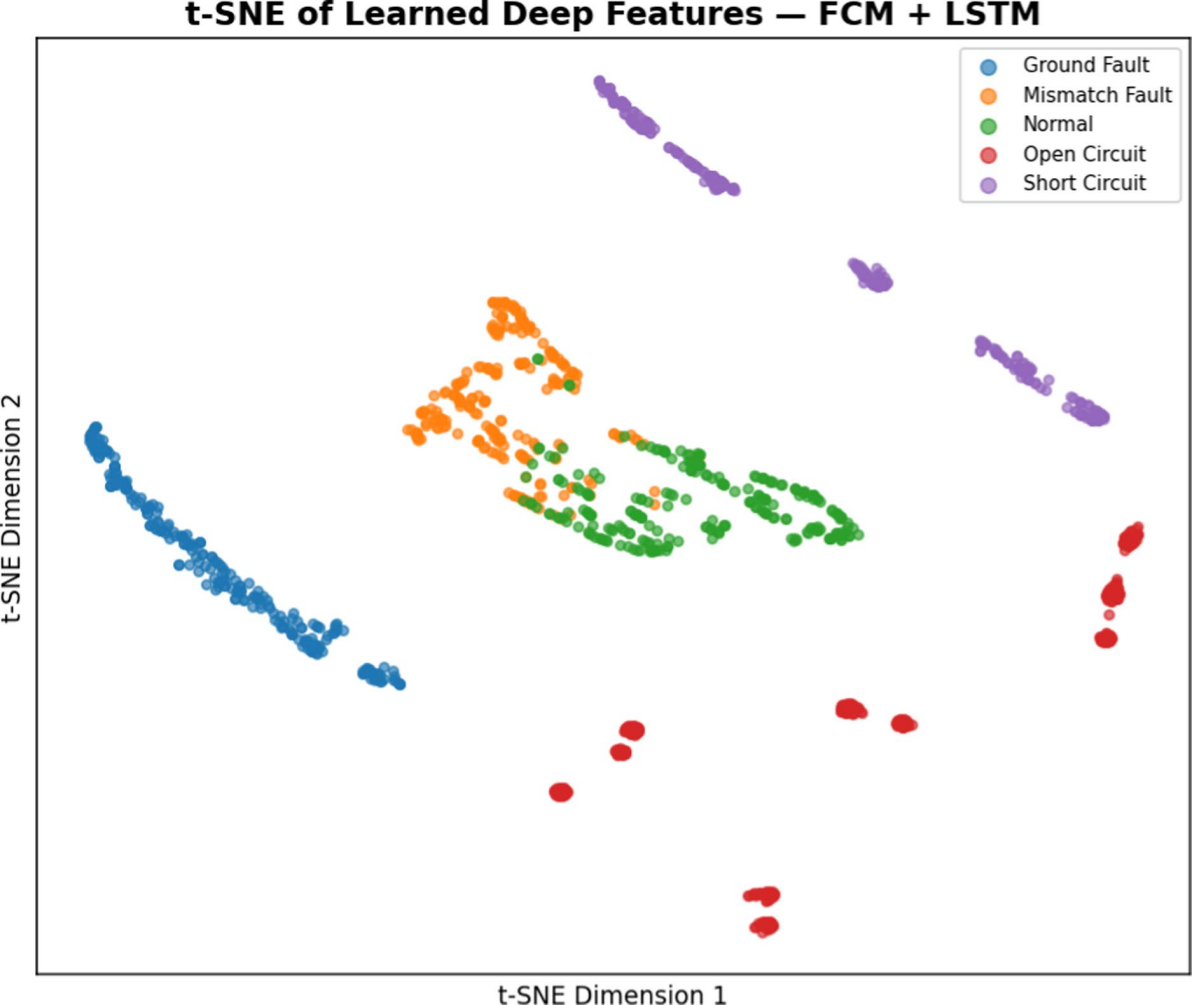
t-SNE visualization of PV fault classes for FCM + LSTM.

**Figure 44:**
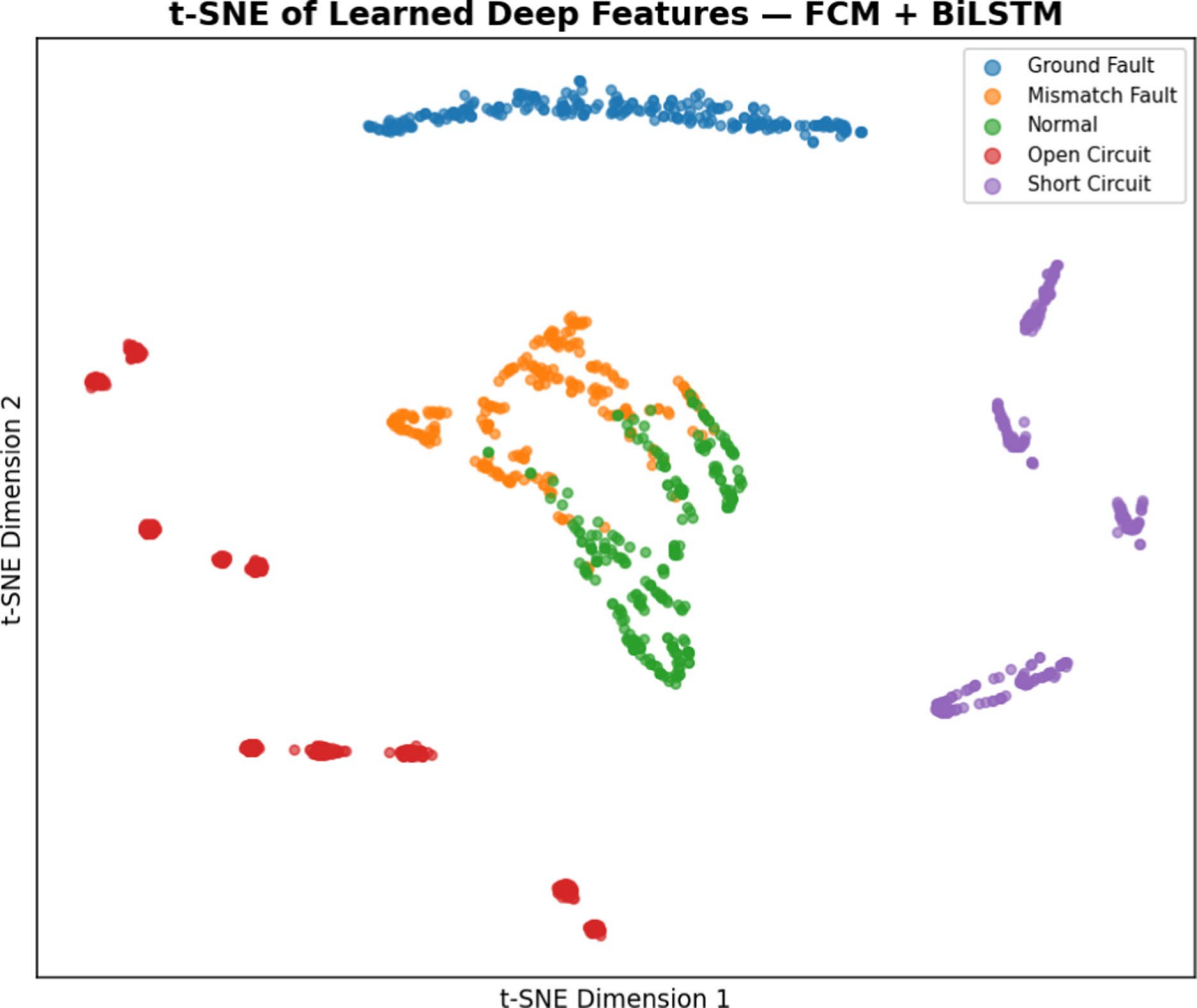
t-SNE visualization of PV fault classes for FCM + BiLSTM.

**Figure 45:**
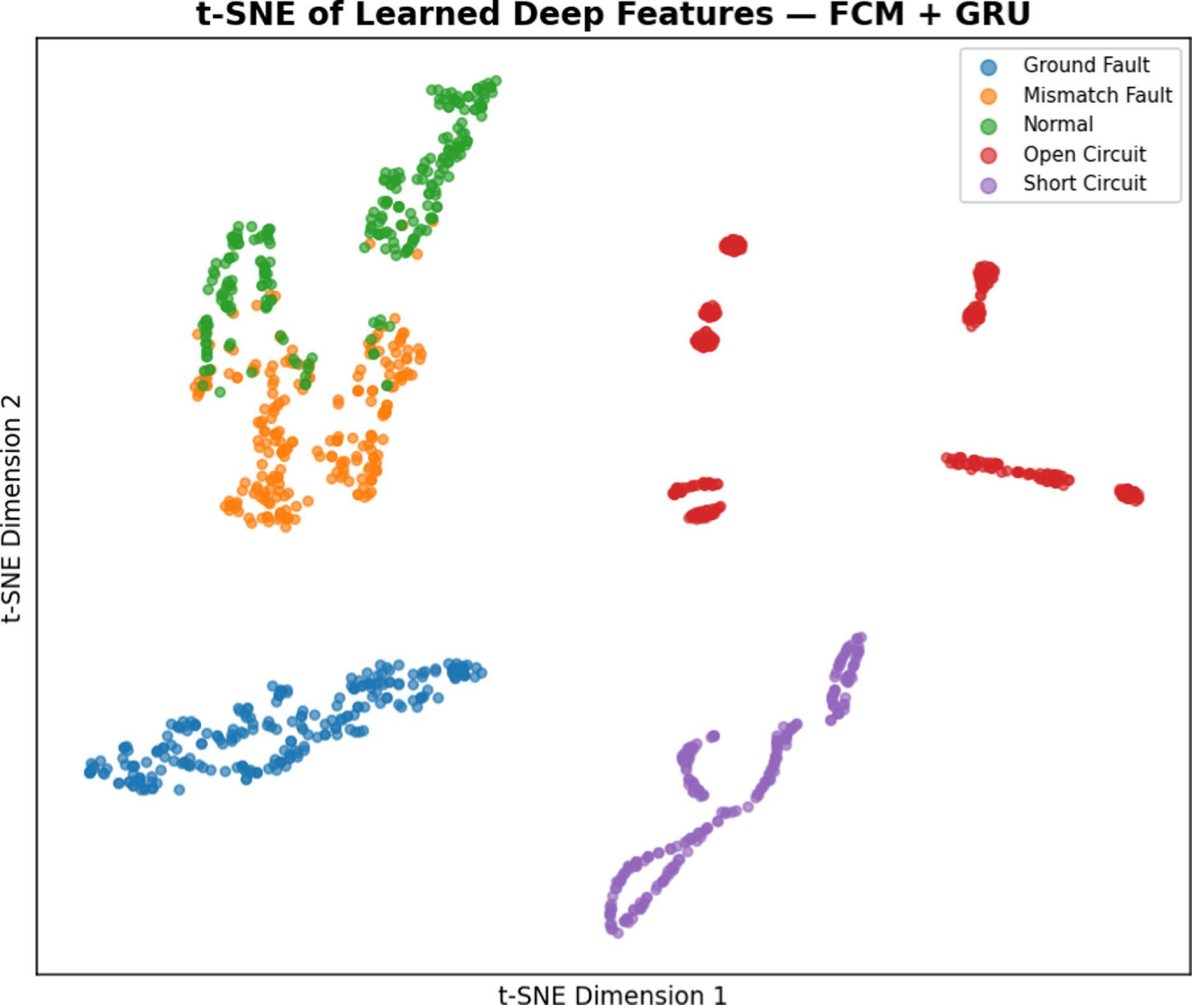
t-SNE visualization of PV fault classes for FCM + BiLSTM.

*k- Boosting rounds, n -training samples, d-maximum depth of each DT.

t-SNE (t-distributed Stochastic Neighbor Embedding) is a nonlinear dimensionality reduction method preferred to visualize high-dimensional feature representations in a two-dimensional space. t-SNE visualization of PV fault classes for the FCM + MLP, FCM + LSTM, FCM + BiLSTM, and FCM + GRU models are shown in Figures 42–45. When compared with other DL models, the t-SNE visualization shows that, the FCM + GRU achieves the best feature representation by producing compact and well-separated clusters with minimal overlap. This demonstrates its superior ability to learn discriminative features and accurately differentiate various fault classes.

**Figure 46:**
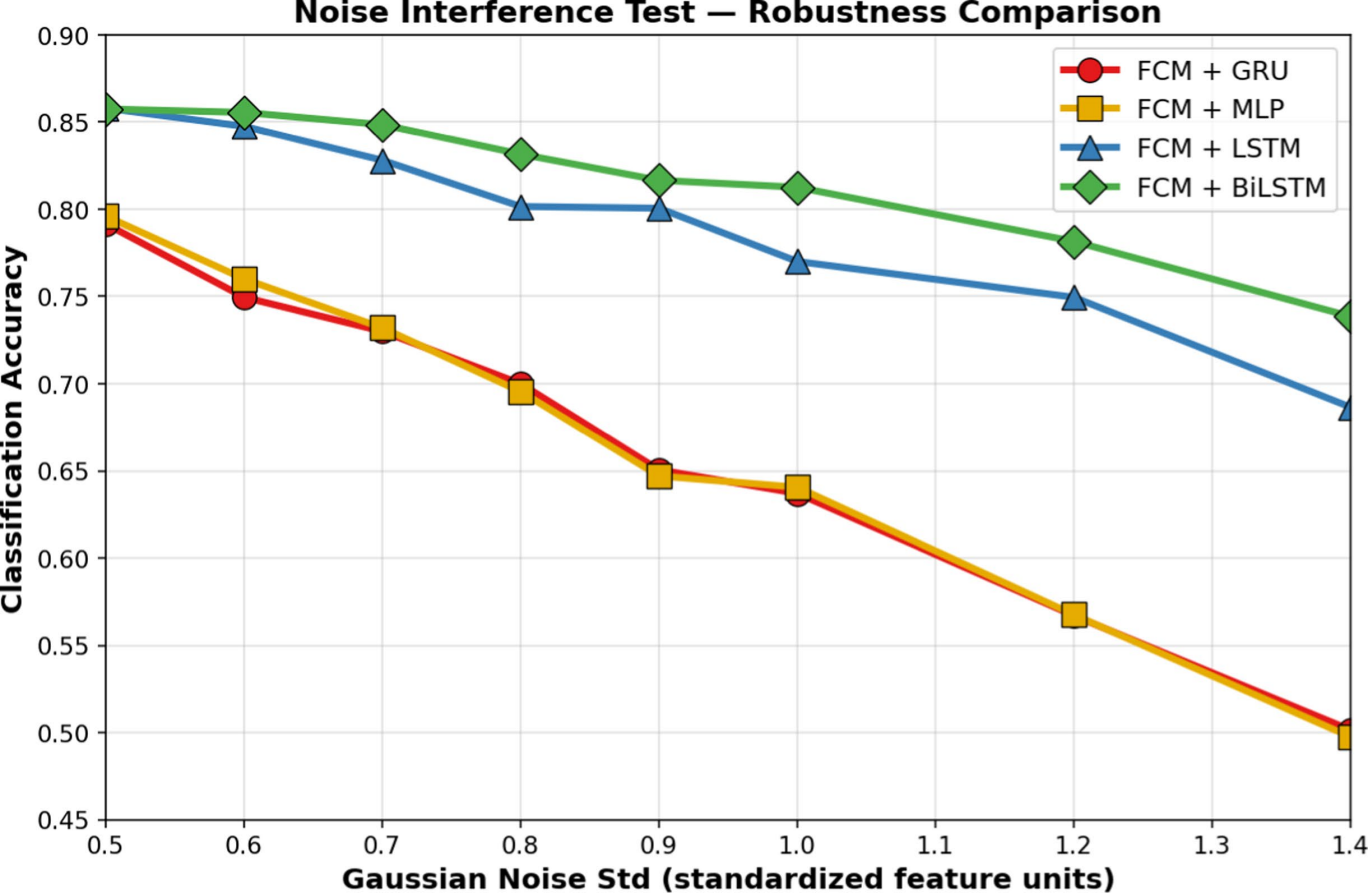
Noise interference test of proposed DL models.

To evaluate the robustness of the proposed framework, a noise interference test was conducted by adding zero-mean Gaussian noise with varying standard deviations to the standardized test dataset. The noisy feature vectors were subsequently processed through the FCM + DL classification framework, and the classification performance was evaluated at each noise level. As shown in Figure 46, all four DL models preserved the classification accuracy under low-to-moderate noise levels, with only a gradual degradation in performance as the noise intensity increased. These findings confirm that the proposed FCM-based DL framework is robust to measurement noise and suitable for reliable PV fault classification under various operating conditions.

## 6 Discussion

The proposed FCM-based hybrid models for PV fault classification with existing works in shown in Table 7. All models have obtained higher classification accuracy, showing the suitability of integration between FCM clustering and ML and DL methods. It is noted that all models successfully differentiate the critical faults, which have GF, OC and SC, respectively, with perfect accuracy, while differentiating between Normal and Mismatch Fault becomes difficult because of overlapping characteristics in these two types of faults. The proposed approach is different from the conventional approaches because it incorporates clustering and more than one learning model to represent the features and improve the fault classification performance under all the major PV fault conditions. Although the integration of FCM raises the computational complexity due to an additional clustering stage, it improves the reliability and decreases the misclassification faults, and makes the proposed system suitable for a practical real-time PV monitoring system. Some existing approaches report higher accuracy; however, such performance may be influenced by overfitting, whereas the proposed method emphasizes generalization and consistent performance across all classes. The proposed approach demonstrates more consistent class-wise performance across all fault categories and improved robustness under varying operating conditions. Also, many existing studies focus on limited fault categories, ideal environmental conditions, or standalone classifiers; the proposed framework considers multiclass photovoltaic fault diagnosis under varying operating conditions. The proposed model was then tested for performance under different weather conditions, such as variations in irradiance and temperature. The obtained results reveal that the model has a robust performance to the environmental variations and maintains consistent classification accuracy in the range of operation considered.

**Table 7:** Proposed work comparison with existing works.

Ref. No.	Fault assessment	OC	SC	GF	MF	ML / DL techniques used	Accuracy	Outcomes	Limitations
Labiod et al. (2025)		✓	✓	✓		Harris Hawks Optimization-XGBoost	99.49%	Improved PV diagnostics using AI integration	Limited fault coverage
Saravanan et al. (2024)		✓	✓	✓		Binary firefly Optimization + ML	100%	Fault detection under shading conditions	Overfitting issues
Khalil et al. (2020)		✓	✓		✓	Model based fault detection	Not Reported	Detailed analysis of PV fault behavior	No experimental validation
Rahman et al. (2021)		✓	✓	✓	–	Measurement-based methods	99%	Accurate OC fault detection	No experimental validation
Punitha et al. (2024)		✓		✓	✓	Efficient Net	97.24%	Real-time fault detection with IoT	Computational complexity
Ramachandran et al. (2025)		✓			✓	CNN	94%	DL based fault detection using RGB images of PV panels	Requires large dataset
Khandeparkar et al. (2025)	✓	✓	✓	✓	✓	RF, SVM, DT, NB, XGBoost	98%	High accuracy ML-based fault classification	Limited temporal analysis
Kataria et al. (2026)				✓	✓	ANN, SVM, DT, RF	98.33%	Effective fault detection using ML	Dataset dependency
Haque et al. (2019)					✓	Snake Optimizer algorithm	Not Reported	PV array-based reconfiguration	Validated for 3*3 PV array
Proposed Work	✓	✓	✓	✓	✓	FCM + MLP, LSTM, GRU, BiLSTM	91.13%	Hybrid clustering + DL for fault classification	Misclassification between MF and Normal

## 7 Conclusion

In this paper, a hybrid fault classification framework integrating FCM clustering with DL models such as MLP, LSTM, BiLSTM and GRU is proposed. The incorporation of FCM improved feature representation by extracting underlying data patterns. Among the investigated models, the FCM + GRU architecture achieved the highest classification accuracy along with balanced precision, recall, and F1-score values. The superior performance of the GRU model can be attributed to its efficient gated memory mechanism and lower computational complexity, enabling better learning of non-linear fault characteristics while maintaining stable convergence behavior. The results confirm the effectiveness of the proposed system in detecting the major electrical PV faults, while also emphasizing the difficulty in separating MF faults from normal operating circumstances due to their similar characteristics. The limitation of this study is that the proposed model was evaluated for only four major fault categories, such as OC, SC, GF and MF. Future work will extend the framework to compound and intermittent fault conditions. Furthermore, the advanced attention-based DL models will be developed to further improve the classification accuracy and robustness of the proposed system. Furthermore, the proposed system can be practically implemented in grid-connected and standalone PV systems. It can be used as an addition to the existing inverter or monitoring devices to continuously monitor the sensor data and identify faults at an early stage. This allows for better reliability of the system, less downtime, and supports predictive maintenance in the PV system.

## Data Availability

The raw data supporting the conclusions of this article will be made available by the authors, without undue reservation.
